# High-Throughput Crystallography Reveals Boron-Containing Inhibitors of a Penicillin-Binding Protein with Di- and Tricovalent Binding Modes

**DOI:** 10.1021/acs.jmedchem.1c00717

**Published:** 2021-07-31

**Authors:** Hector Newman, Alen Krajnc, Dom Bellini, Charles J. Eyermann, Grant A. Boyle, Neil G. Paterson, Katherine E. McAuley, Robert Lesniak, Mukesh Gangar, Frank von Delft, Jürgen Brem, Kelly Chibale, Christopher J. Schofield, Christopher G. Dowson

**Affiliations:** † School of Life Sciences, University of Warwick, Coventry CV4 7AL, U.K.; ‡ Diamond Light Source Ltd, Harwell Science and Innovation Campus, Didcot OX11 0DE, U.K.; § Department of Chemistry and the Ineos Oxford Institute of Antimicrobial Research, Chemistry Research Laboratory, 12 Mansfield Road, Oxford OX1 3TA, U.K.; ∥ Drug Discovery and Development Centre (H3D), University of Cape Town, Rondebosch 7701, South Africa; ⊥ Structural Genomics Consortium (SGC), University of Oxford, Oxford, U.K.; # Department of Biochemistry, University of Johannesburg, Auckland Park 2006, South Africa; ¶ Research Complex at Harwell, Harwell Science and Innovation Campus, Didcot OX11 0FA, U.K.; ∇ South African Medical Research Council Drug Discovery and Development Research Unit, Department of Chemistry and Institute of Infectious Disease and Molecular Medicine, University of Cape Town, Rondebosch 7701, South Africa

## Abstract

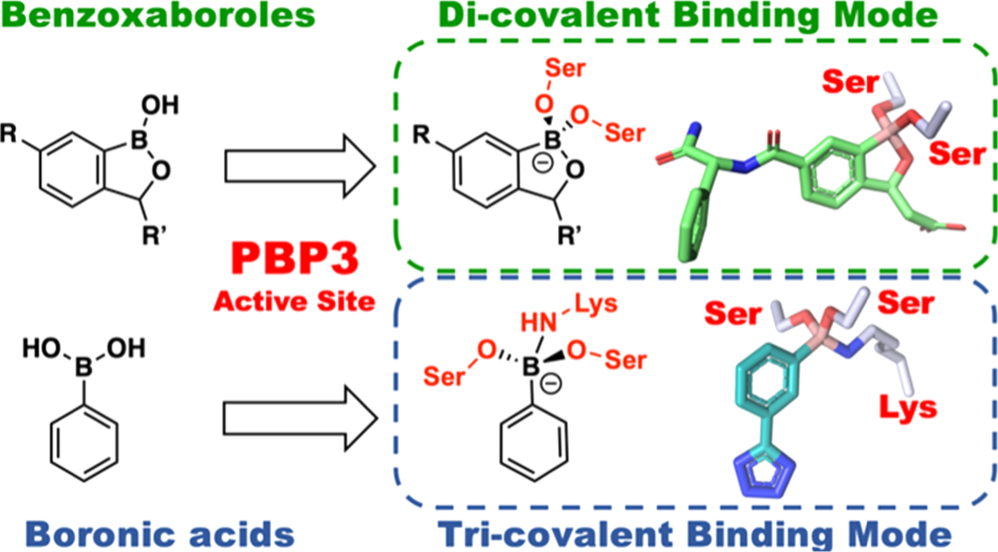

The effectiveness of β-lactam antibiotics is increasingly compromised by β-lactamases. Boron-containing inhibitors are potent serine-β-lactamase inhibitors, but the interactions of boron-based compounds with the penicillin-binding protein (PBP) β-lactam targets have not been extensively studied. We used high-throughput X-ray crystallography to explore reactions of a boron-containing fragment set with the *Pseudomonas aeruginosa* PBP3 (PaPBP3). Multiple crystal structures reveal that boronic acids react with PBPs to give tricovalently linked complexes bonded to Ser294, Ser349, and Lys484 of PaPBP3; benzoxaboroles react with PaPBP3 via reaction with two nucleophilic serines (Ser294 and Ser349) to give dicovalently linked complexes; and vaborbactam reacts to give a monocovalently linked complex. Modifications of the benzoxaborole scaffold resulted in a moderately potent inhibition of PaPBP3, though no antibacterial activity was observed. Overall, the results further evidence the potential for the development of new classes of boron-based antibiotics, which are not compromised by β-lactamase-driven resistance.

## Introduction

β-Lactam antibacterials, that is, penicillins, carbapenems, monobactams, and cephalosporins, target the penicillin-binding protein (PBP) family of transpeptidases. In Gram-negative bacteria, inhibition of high-molecular mass (HMM) class A and class B PBPs (PBP1a/b, PBP2, and PBP3) is typically lethal.^1,2^ The class B PBP3 is a monofunctional peptidoglycan transpeptidase, which is associated with cell division, where it cross-links stem peptides of polymerized molecules of lipid II to create the peptidoglycan mesh essential for bacterial survival.^3,4^ β-Lactams inhibit PBPs, initially by competing with the D-Ala-D-Ala terminus of the stem peptide substrate to give a non-covalent complex, which then reacts with the active-site catalytic serine [in *Pseudomonas aeruginosa* PBP3 (PaPBP3): Ser294] to give an acyl–enzyme complex which is stable over a biologically relevant timescale.^5^

The effectiveness of β-lactams to treat Gram-negative infections caused by *Escherichia coli*, *Klebsiella pneumoniae*, *Enterobacter cloacae*, *Acinetobacter baumannii*, and *P. aeruginosa* is increasingly compromised by serine- and/or metallo-β-lactamases (SBLs and MBLs, respectively),^6,7^ with >1800 BL variants identified.^7^ Ambler classes A, C, and D BLs are SBLs, while those employing a zinc ion-mediated mechanism are Ambler class B BLs (MBLs).

The ability of penicillins to treat infections is enhanced by their combination with a class A SBL inhibitor (e.g., clavulanate);^6,7,9^ however, the rise of Class A (i.e., *K. pneumoniae* carbapenemases) and class D BLs has compromised this approach. Non-β-lactam-based carbapenemase inhibitors, that is, diazabicyclooctanes (DBOs), for example, avibactam, which inhibits class A, C, and some D BLs;^6,7,10^ and relebactam, which inhibits class A and C BLs,^11−13^ show utility (avibactam and relebactam) and promise (other DBOs) in restoring the clinical efficacy of β-lactams against some resistant strains though they are not potent MBL inhibitors. There is thus a need for new antibiotics to treat a wider spectrum of resistant Gram-negative infections.^14^ The development of antibiotics with novel modes of action avoiding existing resistance mechanisms is time-consuming. An alternative is to identify new chemo types for validated targets and to incorporate new features that limit resistance mechanisms to existing antibiotics.

**Figure 1:**
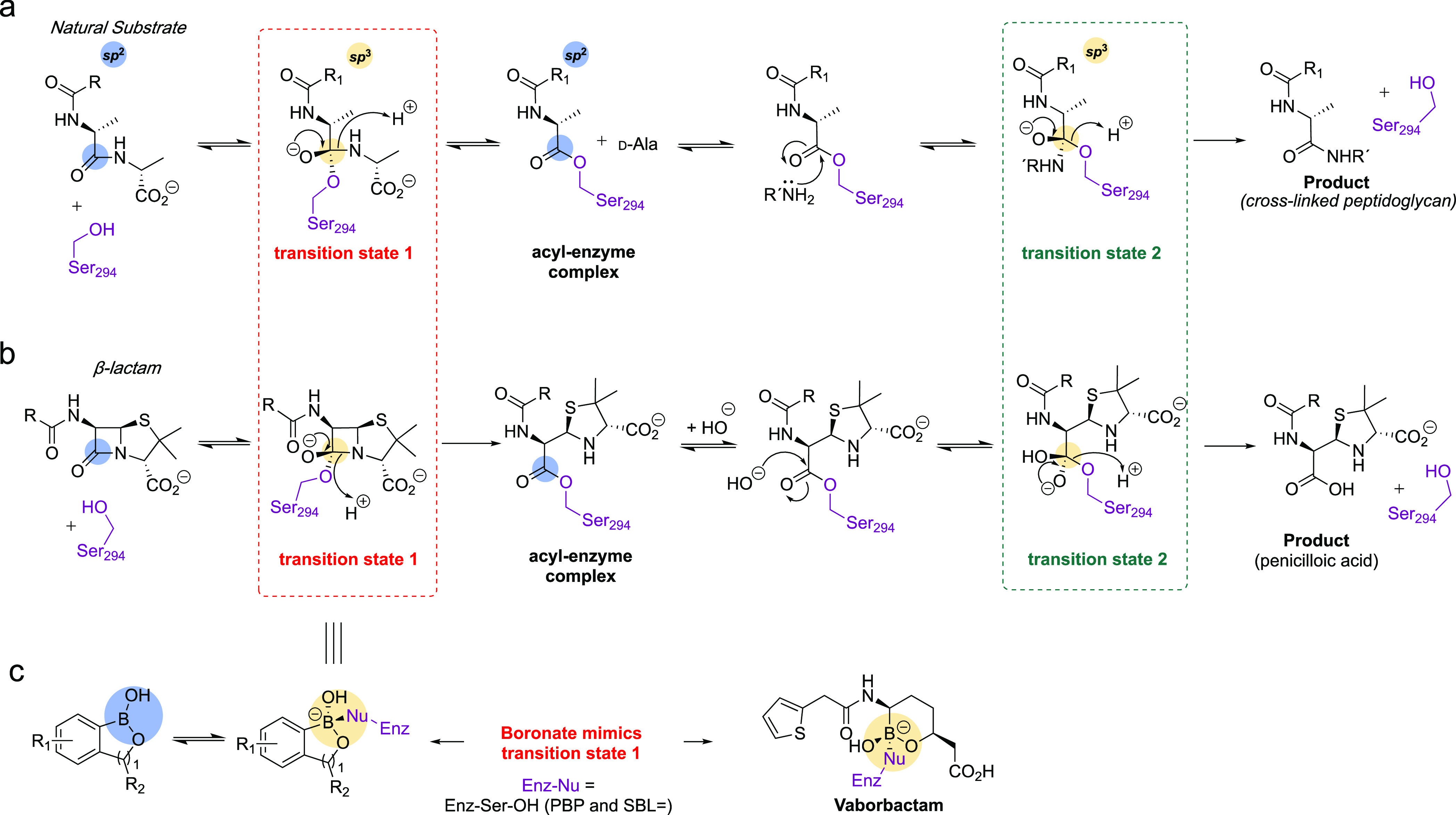
BCIs are proposed to act as mimics of “tetrahedral” transition states arising from enzyme-catalyzed hydrolysis pathways of natural substrates and β-lactams with PBPs. Outline of general mechanisms of (a) transpeptidase reactions catalyzed by a class B PBP, for example, PaPBP3 and (b) the reaction of β-lactam with PBP3, exemplified with a penicillin. Hydrolysis of the acyl–enzyme complex is typically slow in PBPs but is rapid in serine-BL (SBL) catalysis. (c) sp^3^ form of BCIs may mimic the proposed “tetrahedral” transition states and thus bind tightly to the PBP active site. The ability of boron to “morph” between sp^2^ and sp^3^ hybridization states is important in the context of SBL and likely MBL inhibition.^8^

Following the pioneering clinical development of boronic acids as protease inhibitors for multiple myeloma treatment,^15^ interest in boron-containing inhibitors (BCIs) of nucleophilic enzymes has been growing. The ability of boronates to inhibit SBLs has long been known. However, it is only recently that monocyclic (i.e., vaborbactam, which inhibits class A and C BLs^16−18^) and bicyclic boronates (e.g., taniborbactam and structurally related bicyclic boronates, which inhibit members of all four Ambler classes of BLs including MBLs^19−21^) suitable for clinical use/development have been reported. These compounds bind in a manner that mimics the proposed “tetrahedral” transition states in SBL and MBL BL catalysis^8,22^ (Figure 1b). By contrast with the work on SBLs and MBLs, there are relatively limited reports on the interactions of boron-based inhibitors with PBPs.^22−33^ Although there are reports of boronic acids reacting with PBPs,^23,24,26−31,34−36^ and with antibacterial activity,^25^ there are no reports of potent bicyclic boron-based PBP inhibitors/antibacterials in the peer-reviewed literature. The development of dual-action PBP/BL boron-based inhibitors is also of interest. The bicyclic BCI scaffolds are of particular interest in part because their rigid nature compared to acyclic inhibitors may make it easier to develop selectivity for bacterial PBP and BL targets over nucleophilic human enzymes. Work with the DBO scaffold, which was originally developed for SBL inhibition but which was subsequently developed for antibacterial use,^37−39^ suggests that bicyclic boron-based PBP inhibitors might be analogously developed.

In addition to acting as “transition-state” analogues of BLs, crystallography reveals the potential for BCIs to undergo unexpected reactions, for example, formation of a tricyclic structure with the NDM-1 MBL^19^ and of a tricovalent binding mode with d,d-peptidases.^27^ We are interested in exploring the reactions of boron-based compounds with PBP inhibitors, with a view of enabling them as non-β-lactam-based inhibitors that are not susceptible to BLs.

Here, we report the use of high-throughput protein crystallography^40^ to investigate the binding modes of a fragment library enriched with boron-based compounds to PaPBP3. The extensive structural results reveal that different types of potential boron-based inhibitors react differently with PBPs, in particular boronic acids react to form tricovalent complexes, while benzoxaboroles form dicovalent complexes.

## Results

### X-ray Fragment Screen

Our previous attempt at high-throughput (XChem) fragment screening with crystals of PaPBP3 with a diverse library of >1300 fragments yielded only a single, covalently reacted hit, a much lower hit rate than typically expected for such screens.^41^ We therefore elected to focus on a covalent fragment library enriched with boron-based compounds, given their demonstrated reactivity toward serine nucleophiles.^42^ In total, 262 compounds (most from Enamine’s “Serine focused Covalent Fragments” library^43^), of which 152 were boron-containing compounds, were tested. For comparison, other electrophilic compounds including epoxides and sulfonyl fluorides and vaborbactam^16−18^ (a monocyclic boronate SBL inhibitor, which reacts in a monocovalent manner^16^) were also included. The library was screened against PaPBP3 crystals (Figure S4) at both pH 6 and pH 8 because BCIs can interact differently with proteins depending on the pH.^44^ Soaking of crystals, harvesting, and data collection were completed within 24 h. Thirty-four boron-containing fragments (boronates and benzoxaboroles) and vaborbactam were determined to be “hits” with various levels of electron density observed at the active site. The fragments showed a clear pattern of either tricovalent^27^ or dicovalent bonding depending on whether the compound was based on a boronate or a benzoxaborole scaffold.

Structures of boronates **1** and **2** with PaPBP3 show the boron atom is sp^3^-hybridized and tricovalently bonded to Ser294 (the catalytic serine), Ser349 (from the conserved **S**xN motif), and Lys484 (in the **K**S(T)G motif). The reaction of Lys484 is notable and may in part reflect a low p*K*_a_ for this residue. Previous studies with PBPs have proposed the presence of a low p*K*_a_ lysine residue (generally the equivalent residue to Lys297) in their active sites.^29,46−48^

**Figure 2:**
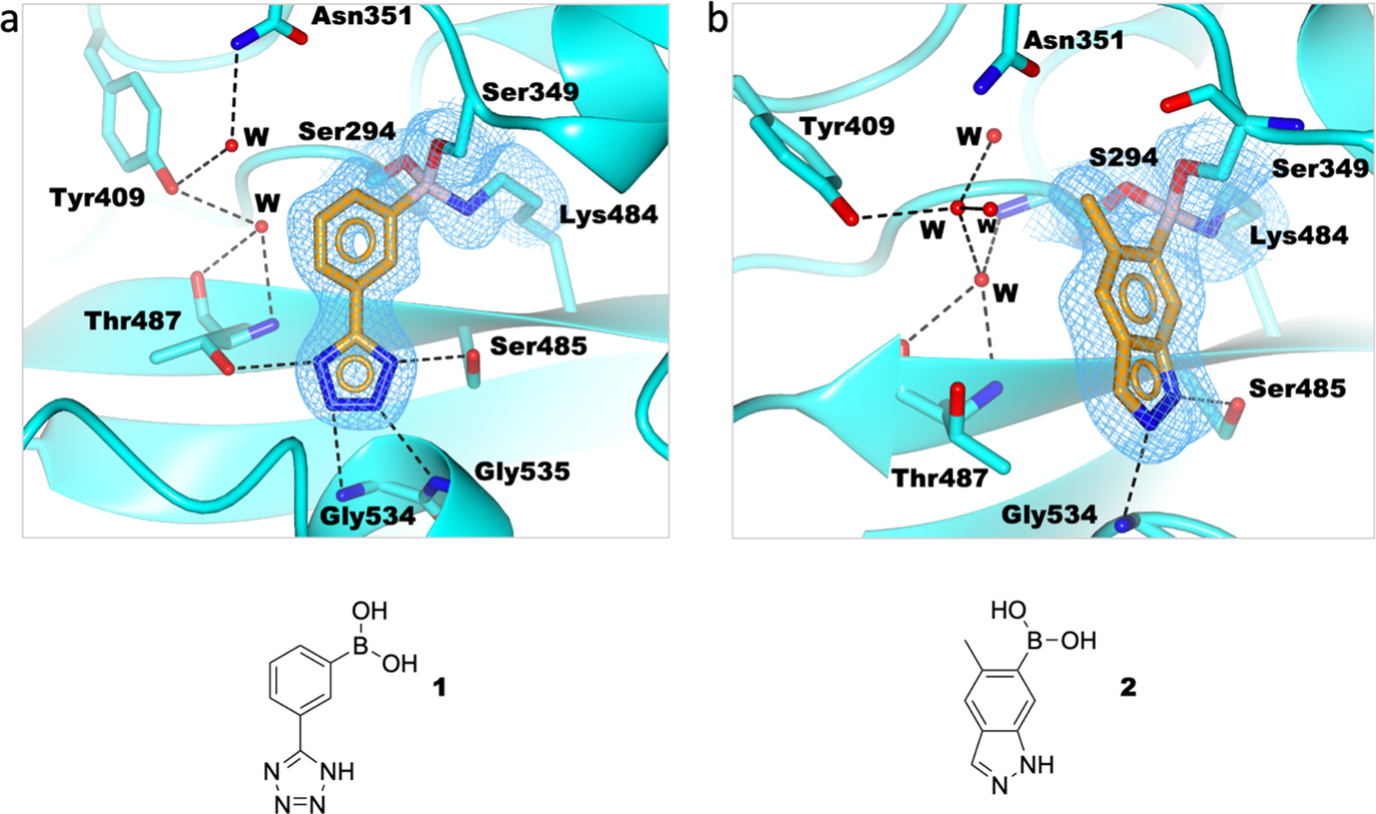
Boronates **1** and **2** react with PaPBP3 in a tricovalent manner. Boronates (a) **1** and (b) **2** react with Ser294, Ser349, and Lys484 (PDB: 7ATM and 7ATO respectively). A dimethyl sulfoxide molecule at the active site in both structures is not shown for clarity (see Figure S10). Hydrogen bonds are shown as black dashed lines. Unbiased omit Fo-Fc maps are shown (light blue mesh) for the ligand and covalently attached side chains (contoured at 1σ), as calculated by “comit” in the ccp4 suite.^45^

The phenyl rings of **1** and **2** occupy similar, but distinct, regions at the active site. Water occupies the oxyanion hole that is occupied by the β-lactam-derived carbonyl of PBP inhibitors (Figure 4 and Figure S5). The tetrazole of **1** makes hydrogen-bonds to the backbone NHs of Gly534 and Gly535 and is positioned to hydrogen bond with the Thr487 and Ser485 side chains (Figure 2). The imidazole of **2** also forms a hydrogen bond with the backbone NH of Gly535 and the Ser485 side chain. The direct hydrogen-bonds to Gly534 and Gly535 are unusual for PBP3 inhibitors that occupy the carboxylic acid binding pocket, that is, the pocket binding the C-3 penicillin carboxylate (Figure S5). These structures therefore provide support for the future design of PBP3 inhibitors incorporating either weakly acidic non-carboxylates or neutral groups^49^ that interact with the “acid binding pocket” of PBP3, which is typically occupied by the C-3 carboxylate of β-lactams (penicillin nomenclature) (Figure S5).

A similar tricovalent binding mode is reported for an alkyl boronate bound to a d,d-peptidase from *Actinomadura* sp. R39 (PDB: 3ZVT)^27^ though our work represents the first time these binding modes have been observed with a HMM PBP. Notably, the boron atoms of both structures are positioned similarly within the active site and the side chains of both nucleophilic serines and lysine align closely, independent of the nature of the boron-bonded functional group, that is, alkyl versus phenyl.

**Figure 3:**
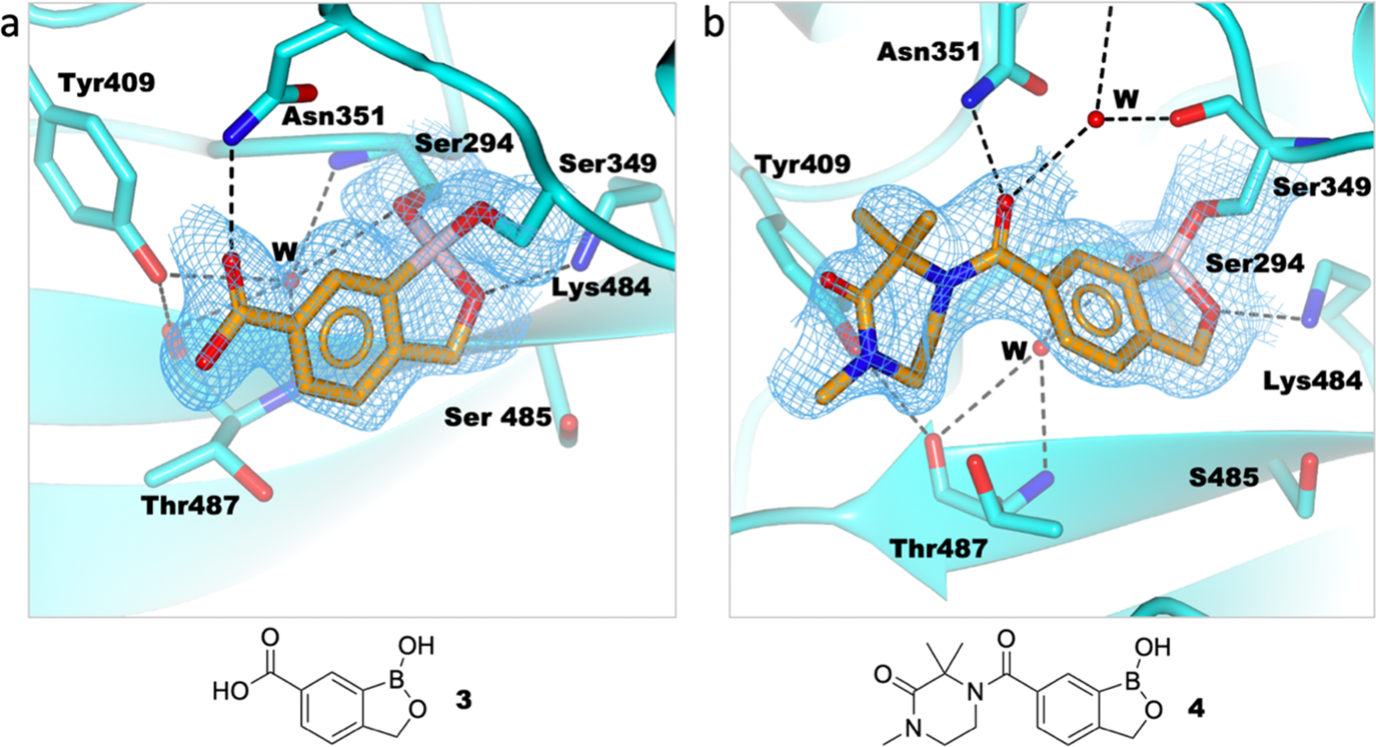
Benzoxaboroles react with PaPBP3 in a dicovalent manner. (a) **3** and (b) **4** react covalently with Ser294 and Ser349 and hydrogen bond with Lys484 (PDB: 7ATW and 7ATX, respectively). Hydrogen bonds are shown as black dashed lines. Unbiased omit Fo-Fc maps (light blue mesh) are shown for the ligand and covalently attached residue side chains (contoured at 1σ), as calculated by “comit” in the ccp4 suite.^45^

The screen also revealed that benzoxaboroles bind to the PBP active site, but with the boron dicovalently bound to Ser349 and Ser294 and a hydrogen bond via its “endocyclic” oxygen to Lys484. Both **3** and **4** form hydrogen bonds to the side chain NH_2_ group of Asn351. Like the phenyl boronates, a water molecule occupies the oxyanion hole and forms hydrogen bonds to the backbone NH of Ser294 and Thr487. In the structures for **3** and **4**, Tyr409 is hydrogen-bonded to the backbone carbonyl of Thr487 (Figure 3).

### Hit Expansion Design and Synthesis

**Figure 4:**
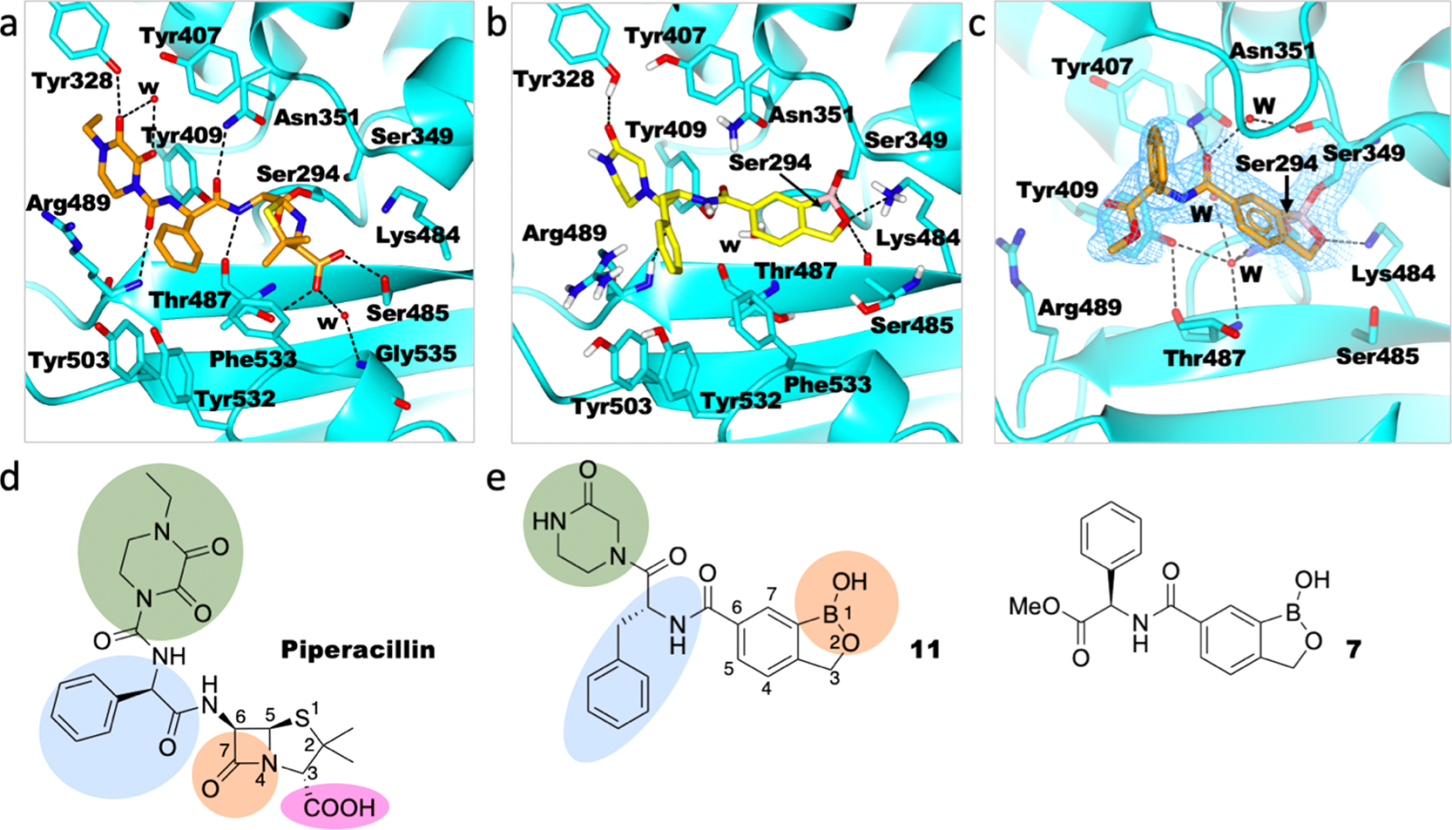
Binding mode of PaPBP3 inhibited by piperacillin compared with those for benzoxaboroles **7** and **11**. (a) PaPBP3 inhibited by piperacillin (PDB: 6R3X);^51^ (b) predicted binding mode of **11** as determined by docking, showing how groups 1 and 2 may engage in the same manner as analogous groups in piperacillin; (c) binding mode of **7** complexed with PaPBP3 (PDB: 7AU0). Hydrogen bonds are shown as black dashed lines. An unbiased omit Fo-Fc map is shown (light blue mesh) of the ligand and covalently attached residue side chains (contoured at 1σ), as calculated by “comit” in the ccp4 suite.^45^ (d) Structure of piperacillin colored according to its functional groups: β-lactam (orange), C-3 carboxylate (pink), group 1 (blue), and group 2 (green); (e) benzoxaborole **11** was designed to mimic the piperacillin binding mode. Colors match analogous groups within piperacillin which were hoped would engage the same parts of the protein in the case of **11**.

Due to the chemically interesting nature of their reaction with the PBP active site, we investigated the potential of benzoxaboroles for PBP inhibition. The PaPBP3/**3** complex structure, along with reported structural studies on β-lactams,^50,51^ was used to design a set of 6-substituted amides incorporating the features of the piperacillin C-6 side chain, that is, d-phenylglycine and diketopiperazine moieties; for synthetic simplicity, a ketopiperazine was used to mimic the diketopiperazine. Ideas were evaluated using docking into the PaPBP3 piperacillin binding site (Figure 4).

**Scheme 1:**
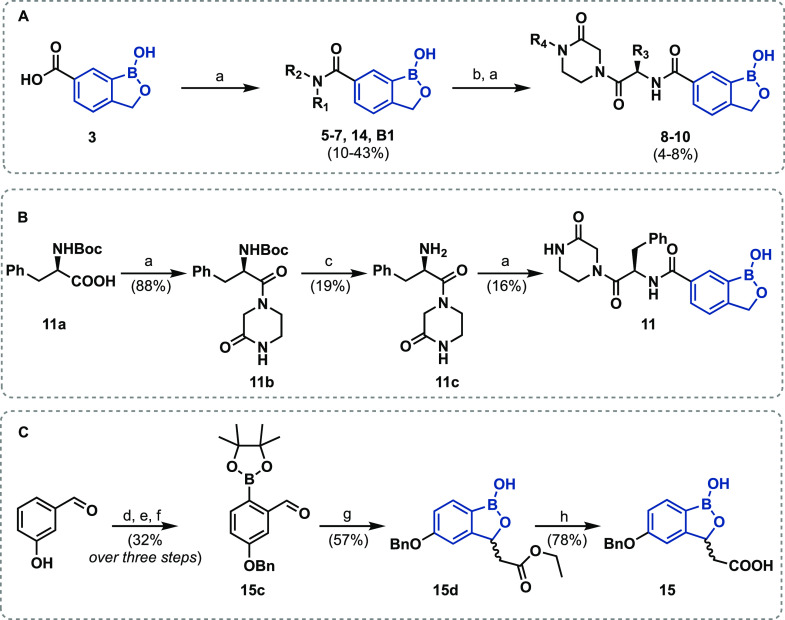
Synthesis of Benzoxaborole Derivatives (A) **5–10** and **14**, (B) **11**, and (C) **15** Reagents and conditions: (a) 1,1′-carbonyldiimidazole, *N*,*N*-dimethylformamide (DMF), 40 °C, 4–16 h; (b) LiOH·H_2_O, 1,4-dioxane/H_2_O (3:1), 40 °C, 60 min; (c) HCl (4 M soln. in 1,4-dioxane), CH_2_Cl_2_, rt,16 h; (d) Br_2_, CH_2_Cl_2_, rt, 18 h; (e) benzyl bromide, K_2_CO_3_, DMF, rt, 4 h; (f) bis(pinacolato)diboron, Pd(dppf)Cl_2_, KOAc, dioxane, 80 °C, 12 h; (g) EtOAc, LDA (1 M solution in THF), −78 to −20 °C, 2 h; (h) LiOH·H_2_O, THF/H_2_O (1:1), rt, 2 h. Note that low isolation yields for substituted benzoxaboroles (e.g., **8–10**) in part reflect significant losses during purification on the silica gel and provide scope for further optimization. For **B1**, R_1_ = H and R_2_ = CH(R_3_)CO_2_Me. The complete structures of **5**–**7**, **14**, and **8**–**10** are shown in Table 1. Dppf: 1,1′-bis (diphenylphosphino)ferrocene; Boc, *tert*-butoxycarbonyl; Bn, benzyl.

Benzoxaboroles **5-11** with a C-6 acylamino side chain were synthesized (Scheme 1) and structures of their PaPBP3 complexes solved. As demonstrated by comparison of Figure 4c,e, despite their covalent reaction with Ser294 and Ser349, **5**–**11** generally failed to engage the active site as proposed and did not show inhibition (Table 1). Analogues **12**, **13** (from Wuxi AppTec), and **15** (Scheme 1C) with a C-3 carboxylic acid group were then synthesized. The C-3 carboxylate group was modeled to align with the analogous C-3 carboxylate present in penicillins (pink in Figure 4), with the aim of improving the affinity.

**Table 1:**
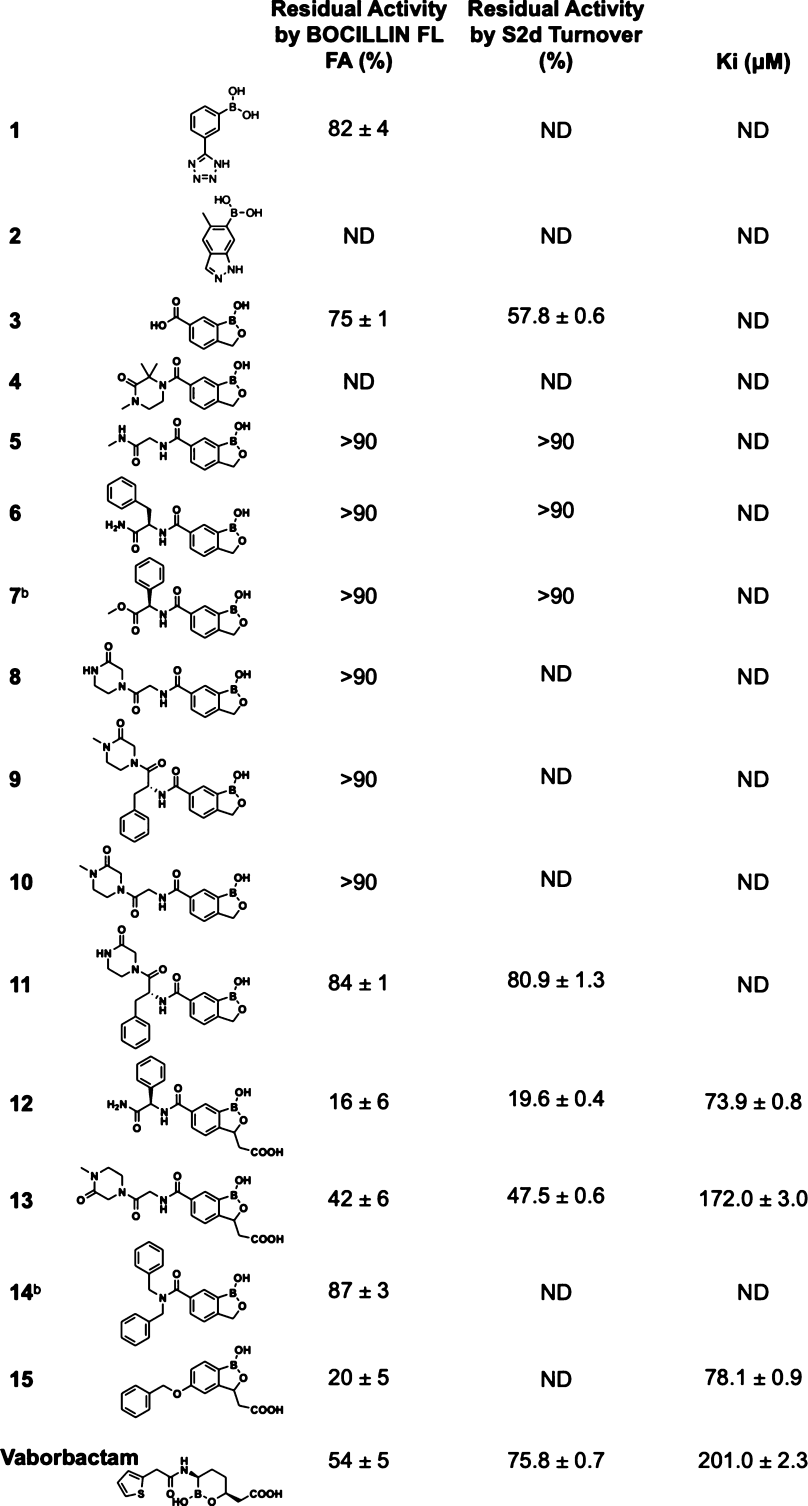
Residual Activities and *K*_i_s of Compounds Tested in the BOCILLIN FL Competition Assay for Binding to PaPBP3 and Residual Activities Measured in S2d Turnover Assays^a^

a For both BOCILLIN FL FA and S2d assays, residual activities in the presence of a 1 mM inhibitor are a percentage of the activity of the untreated control. Errors are standard errors (*n* = 3) from independent measurements.

b Residual activity measured at 100 μM due to solubility issues at 1 mM. *K*_i_, calculated with 11 concentrations of each compound and determined using global fitting in Kintek Global Explorer.^52,59^ ND, not determined.

To explore alternatives to amino acid-derived benzoxaboroles, **14** and **15** were synthesized (Scheme 1). Once again, a dicovalent reaction of the boron with both Ser294 and Ser349 was observed. The phenyl groups within the side chains of both these compounds are situated in a region close to where the reacted piperacillin phenyl binds (PDB: 6R3X)^51^ (Figure S8).

### Inhibition Assays

**1**–**15** were screened for PaPBP3 inhibition at 1 mM using an established fluorescence anisotropy (FA) assay.^52^ For selected compounds, inhibition of hydrolysis of the thioester substrate analogue **S2d**(4,24,53,54) was measured, with good correlation between the results with the two assays (Table 1). Most compounds manifested minimal inhibition despite clear evidence of binding *in crystallo*. Pre-incubation of **12** with PaPBP3 (0, 30, or 60 min) prior to assay initiation had no significant effect on inhibition (data not shown). *K*_i_ values were determined using the BOCILLIN FL assay for the four compounds with the lowest residual activities (**12**, **13**, **15**, and vaborbactam), of which **12** was the most potent with a *K*_i_ of 73.9 ± 0.8 μM (Table 1). **12** was also tested against purified PBP3s from *E. coli*, *A. baumannii*, and *Haemophilus influenzae*, and PBP2 from *Neisseria gonorrhoeae* (equivalent to PBP3)^55,56^ (Table 2). Given the sequence (Figure S6) and structural (Figure S7) similarities of the studied PBP3 variants, the apparent variation in their activity is interesting, possibly indicating a degree of selectivity.

**Table 2:** Inhibition Properties of Benzoxaborole 12 against Various PBP variants^a^

protein tested	residual activity by BOCILLIN FL FA (%)
PBP3 from *P. aeruginosa*	16 ± 6
PBP3 from *H. Influenzae*	32 ± 3
PBP3 from *A. baumannii*	73 ± 3
PBP3 from *E. coli*	>90
PBP2 from *N. gonorrhoeae*	>90

a Residual activities (treatment with **12** (1 mM) for 1 h, the activity determined by BOCILLIN FL competition assay) are given as a percentage of the untreated control, with errors as standard deviations of three technical replicates. Note that the *N. gonorrhoeae* PBP2 was a transpeptidase-only construct.

To investigate the reversibility of the reaction, we used the colorimetric substrate nitrocefin, which acylates PaPBP3 and then rapidly deacylates, with a concurrent color change.^22,57,58^ Upon twofold dilution of the assay, the half–maximal inhibitory concentration (IC_50_) value relative to the uninhibited control was doubled, indicative of rapid (equilibrium established in <30 s) reversibility. The IC_50_ of irreversibly bound ceftazidime was not significantly affected (Figure S3). Consistent with this is the observation that the progress curves can be fit well using a simple reversible, one-step binding model (Figure S2). While the inhibition is weak, these results are consistent with other investigations of boronate binding in HMM PBPs, which typically show IC_50_s in the μM range.^23−25,32,33^

Selected compounds were screened against *E. coli*, *P. aeruginosa*, *H. influenzae*, *A. baumannii*, and *N. gonorrhoeae* and a *P. aeruginosa* strain engineered to remove the outer membrane permeability barrier to investigate their antimicrobial activity (Table S2).^60^ All were ineffective (MICs ≥64 μg/mL). Synergy with piperacillin, a Gram-negative PBP3 specific β-lactam,^61^ was not observed in non-BL-expressing *E. coli* and *P. aeruginosa*, although weak synergy was observed in class C BL-expressing strains (data not shown).

## Discussion

The 10 structures of boron compounds reacted with PaPBP3 (Table S2) reveal three distinct binding modes: (i) monocovalent reaction with Ser294 (Figure 6a), (ii) dicovalent reaction with Ser294 and Ser349 (Figure 6b), and (iii) tricovalent reaction with Ser294, Ser349, and Lys484 (Figure 6c). The observed binding mode depends on the nature of the boron compound, with vaborbactam reacting monocovalently, benzoxaboroles (**3–15**) reacting dicovalently, and phenyl boronates (**1** and **2**) reacting tricovalently. Tricovalent^27^ and monocovalent^22,25−27,29,30^ bonding of boron compounds with PBPs are known, but to our knowledge, this is the first time that benzoxaborole compounds have been shown to bind to a PBP in a dicovalent manner. Although we cannot rule out the possibility that some of our structures are a consequence of crystallization, analogous data were collected at pH 6 and pH 8; the observed covalent binding mode thus does not appear to correlate with the crystallization conditions but is probably related to the form of the warhead (Table S3).

Structures of PaPBP3 with **3**, **4**, **7**, **9**, **12**, **13**, **14**, and **15** show the benzoxaborole core binding in a conserved manner (Figure S8). In the dicovalent benzoxaborole binding mode, the *chi*1 angle of the Ser294 side chain is the same as in the monocovalently piperacillin-reacted structure, but the benzoxaborole and piperacillin-reacted structures have a difference in the *chi*2 angle of ∼80° (Figure S9). These observations reveal a scope for variations in the Ser294 side chain conformation on covalent inhibitor reaction. By contrast, in all our structures, Ser349 is not significantly displaced compared to the β-lactam-reacted structures, for example, piperacillin-reacted PaPBP3 (PDB: 6R3X).^51^ Similarly, in the tricovalent binding mode of **1**, the Ser294 *chi*1 angle rotates by ∼100°(relative to the monocovalently reacted structure) (Figure S9), but the Ser349 and Lys484 side chains are not significantly displaced (with O^γ^ and N^ε^ being displaced <1 Å relative to their positions in the piperacillin-reacted structure (PDB: 6R3X)^51^).

**Figure 5:**
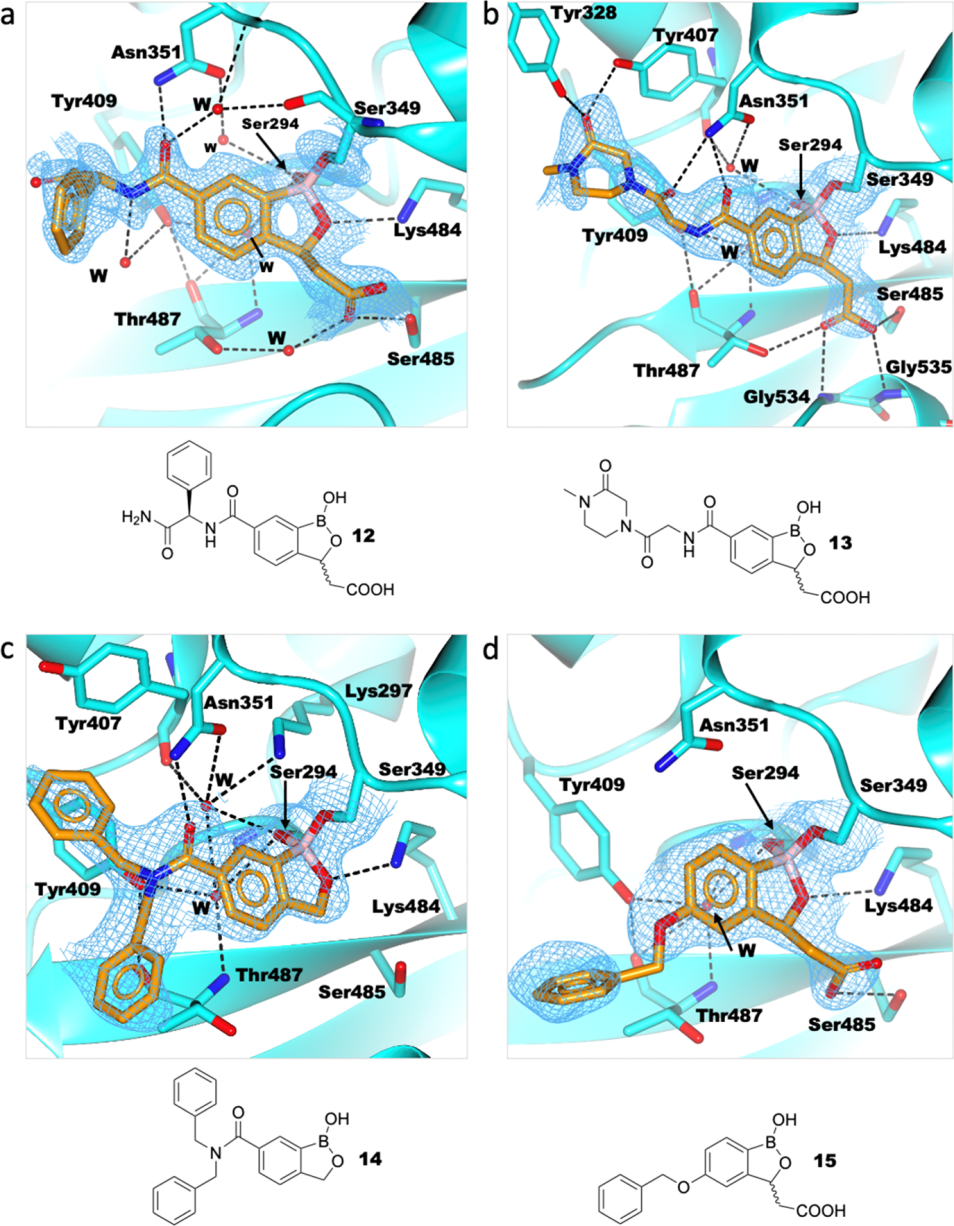
Structures of benzoxaboroles with a C-3 acid group (**12**, **13**, and **15**) and **14** complexed with PaPBP3. (a) PaPBP3/**12** complex (PDB: 7AU1); (b) PaPBP3/**13** complex (PDB: 7AU8); (c) PaPBP3/**14** (PDB: 7AU9); and (d) PaPBP3/**15** complex (PDB: 7AUB). For clarity, only one of the two refined conformations of Tyr409 is shown in (a). Hydrogen bonds: dashed black lines. Unbiased omit Fo-Fc maps are shown (light blue mesh) of the ligand and covalently attached residue side chains (contoured at 1σ), as calculated by “comit” in the CCP4 suite.^45^

It has been suggested that, at least in the context of BL inhibition, binding to the catalytic serine occurs first, followed by (where appropriate) reaction with the other nucleophilic residues.^27^ In the structures where boron reacts in the dicovalent manner (see, e.g., Figure 5), substitution of the strong B–O bond in the five-membered ring of the benzoxaborole^62,63^ by the primary amine of Lys484 is likely unfavorable, explaining why tricovalent binding does not occur.

**Figure 6:**
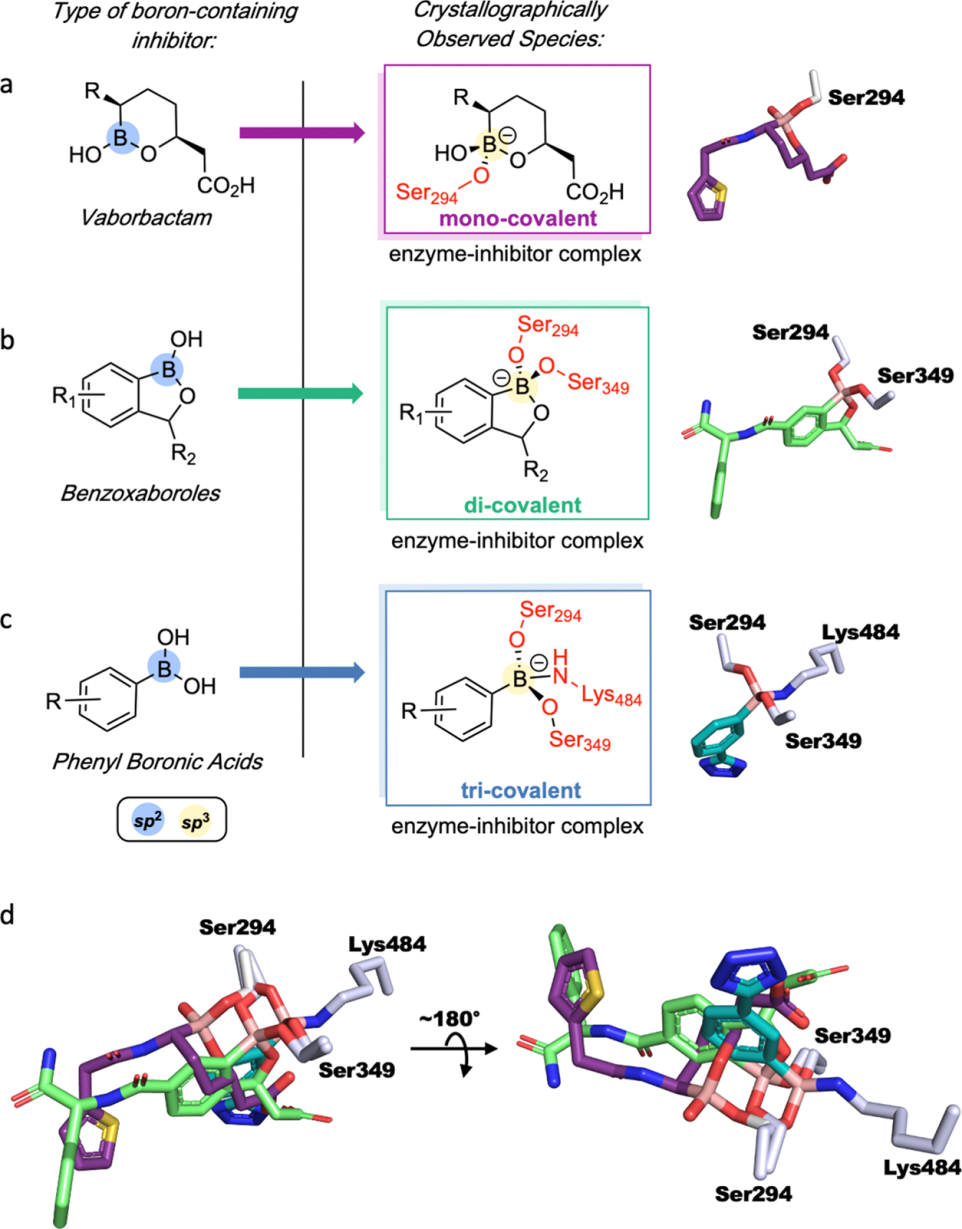
Boron-containing compounds react with a PBP in three distinct modes. The formation of different complexes with exemplary crystallographically observed complexes are shown. (a) Vaborbactam reacts monocovalently with Ser294; (b) Benzoxaboroles (**3**–**15**) bind dicovalently with Ser294 and Ser349; (c) phenylboronic acids (e.g., **1**, **2**, and alkyl boronic acids^27^) bind tricovalently to Ser294, Ser349, and Lys484. The order of nucleophilic residue reactions is unknown; (d) overlay of crystallographically observed states. The position of the boron can be described by rotations of the *chi* angles of the Ser294 side chain. In the monocovalently and dicovalently bound crystal structures [and in the piperacillin-reacted structure (PDB: 6R3X),^51^ the *chi*1 angle of Ser294 is gauche- (∼−60°) relative to the serine amine.^64^ In contrast, in tricovalent mode (e.g., 1), the *chi*1 angle of Ser294 is trans (∼−161°) relative to the amine. There is an 80° difference between the *chi*2 angles of dicovalent mode and monocovalent mode (Figure S9).

Our kinetic data indicate that the equilibrium between PBP and boronate is achieved at least within minutes, supporting a relatively fast, but weak binding model for boronates to PBPs, as suggested previously.^31^ Our method does not allow for determination of binding rates on the seconds timescale, but detailed kinetic analysis has been carried out to investigate the observation of biphasic inhibition curves with some tricovalently binding BCIs.^27^ Zervosen et al. concluded that the biphasic curves were caused by rapid non-covalent association, followed by slow formation of monocovalent, tetrahedral complexes.^27^ The formation of tricovalent complexes was suggested to be in rapid equilibrium with the monocovalent complex (Figure 6c). Boronates that react with PBPs monocovalently are proposed to act as analogues of the tetrahedral transition state (Figure 1);^28−30^ however, the tri- and dicovalent binding modes are less obvious transition state mimics. It remains unclear if the tricovalent binding mode will be important for developing boron-based PBP inhibitors with increased potency, or if it is, at least in part, a crystallographic artifact.

One conclusion from the structural–activity relationships is that the presence of a C-3 acid group increases potency. Comparisons of the affinities of **13** (*K*_i_ = 172.0 ± 3.0 μM) and **10** (residual activity by BOCILLIN FL FA > 90%) demonstrate the benefit of a C-3 acid (Table 1). This result is consistent with the conservation of the C-3 (or equivalent) carboxylate group across most β-lactam PBP inhibitors and studies showing addition of a carboxyl group to benzoxaboroles leads to a 10–100-fold increase in binding affinity for some BLs.^65^

Our structure-based design of boron inhibitors aimed to utilize analogous hydrogen bond interactions to those made by reacted piperacillin (Figure 4). However, the constrained position of the benzoxaborole ring (Figure S8), imposed by the dicovalent bond formation and rigid 6–5 ring system, meant key hydrogen bond interactions, particularly those to the backbone of the β3 strand (Thr487 and Arg489), could not be made by the C-6 amide group (blue in Figure 4d), likely contributing to the lack of inhibition shown for some compounds (**5**–**10**). The position of the C-6 amide group did enable hydrogen bonding with the highly conserved Asn351, a hydrogen bond observed in many β-lactam-reacted PBP complexes (Figure 4a).

Exploration of C-5-substituted benzoxaboroles employing structural insights revealed here could lead to further potency improvements. Thus, for example, while **14** did not inhibit PaPBP3, similar to the other benzoxaboroles without an acid group, one of its benzyl groups is positioned near the D-Phe position of reacted piperacillin and the other is positioned close to key active-site residues. The structure with **15** (*K*_i_ = 78.1 ± 0.9 μM) places the phenyl of the C-5 benzylether benzoxaborole substituent in the same PaPBP3 active-site location as the phenyl rings in piperacillin- and amoxicillin-derived adducts.

Importantly, structures of **1** and **2** demonstrate the potential for non-acid groups to engage the PBP3 acid binding pocket—optimized binding of these groups may be used to combat point mutation-mediated resistance in this region.^51^

Examination of the reported PaPBP3 structures^50,51,66−69^ reveals the β5-α11 loop (residues 528–539, Figure S10) adopts different conformations. The β-lactam-containing PaPBP3 inhibitors (e.g., amoxicillin, aztreonam, ceftazidime, and piperacillin^50,51^) induce formation of a “hydrophobic wall” composed of Tyr533, Phe532, and residues within the β5-α11 loop together with Tyr503.^50^ Meropenem, which lacks a hydrophobic C-6 group, by contrast, does not induce the formation of this “hydrophobic wall” (PDB: 3PBR),^50^ while apo PaPBP3 adopts a third conformation for the β5-α11 loop (Figure S10, PDB: 6HZR).^51^

In most of our PaPBP3 boron-based compound structures (e.g., **12**), the electron density maps in the region of the β5-α11 loop were of insufficient quality to enable residues to be fitted. However, although the observed densities were weak (Figure S10), in the complexes with **1**, **3**, and **13**, unique conformations of the β5-α11 loop were observed. Benzoxaboroles **9**, **11**, **12**, **14**, and **15** were designed to have a phenyl group, binding of which may mimic that of the D-Phe of reacted piperacillin in interactions with the hydrophobic wall (Figure S11). While the phenyl group of **14** and **15** is positioned close to the position of the D-Phe of reacted piperacillin, the hydrophobic wall was not formed on binding, perhaps in part reflecting the poor potency of these compounds.

The conformation of the β3 β-strand is associated with hydrophobic wall formation possibly mediated by inhibitor interactions with Thr487 and Arg489 .^50^ The overall fold of PaPBP3 bound to **12**, which lacks the ketopiperazine terminal substituent, is similar to that in the meropenem-reacted PaPBP3 complex (Figure S12, PDB: 3PBR).^50^ In both these structures, Tyr409 forms a hydrogen-bond to the backbone carbonyl of Thr487, whereas with amoxicillin, aztreonam, ceftazidime, and piperacillin, the inhibitor-derived adducts form a direct hydrogen-bond to the backbone carbonyl of Thr487 (Figure S12). The rigid dicovalent binding mode of the benzoxaborole core (Figure S8) prevents the C-6 amide from directly forming hydrogen-bonds to the backbone carbonyl of Thr48. A structure of PaPBP3 complexed with **13**, which has a ketopiperazine substituent but which lacks a phenyl analogous to that of the D-Phe of piperacillin, has β5-α11 loop and β3 β-strand conformations similar to those observed in the piperacillin-reacted PaPBP3 (Figure S13). Interestingly, both the ketopiperazine of **13** and the diketopiperazine of reacted piperacillin form hydrogen bonds with the O^η^ oxygens of Tyr328 and Tyr407 (Figure S13). Further optimization of the side chains of the benzoxaboroles may be able to better exploit the interactions possible with the β3 strand backbone, Tyr328 and Tyr407, and the hydrophobic wall regions, leading to improved binding.

**Figure 7:**
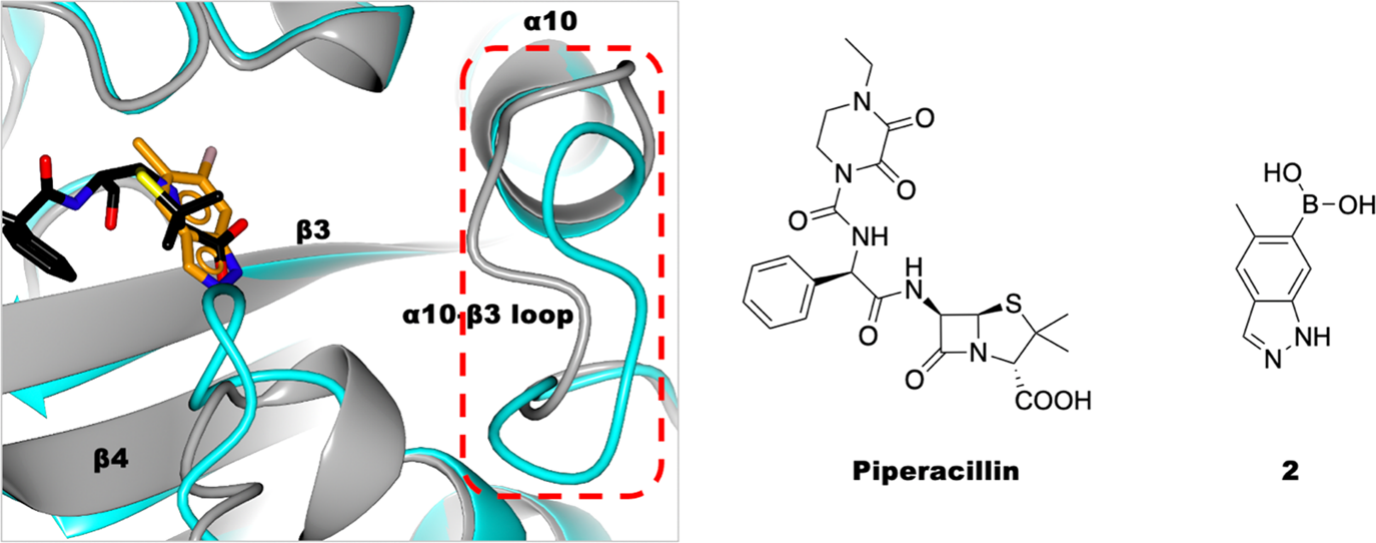
Binding of **2** causes changes in the PaPBP3 active-site conformation. Views of benzoxaborole PaPBP3/**2** (orange, PDB: 7ATO) and piperacillin-reacted PaPBP3 (black, PDB: 6R3X)^51^ (protein backbone colored blue and gray, respectively) are shown. A clear unprecedented difference in the α10-β3 loop conformation (residues 466–473) is observed. See also Figure S14.

Variations in the α10-β3 loop were observed in the PaPBP3/**2** complex (Figures 7 and S14), that is, it adopts a conformation not previously observed in PaPBP3 structures. The largest displacements, relative to the piperacillin-reacted structure, are for residues Gly469, Gly470, and Val471 (7.2, 10.2, and 9.9 Å, respectively). The significance of these movements is unclear. The structure of PaPBP3/**12** was refined with two conformations for Val_476_-Pro-Gly-Tyr-His_480_ of the α10-β3 loop (Figure S15). These structures appear to nicely exemplify how, when combined with high-throughput crystallography, the ability of boron-based compounds to interchange between different forms (including sp^2^ and sp^3^ forms) during their reactions with proteins can be employed to reveal otherwise latent conformations.

**Figure 8:**
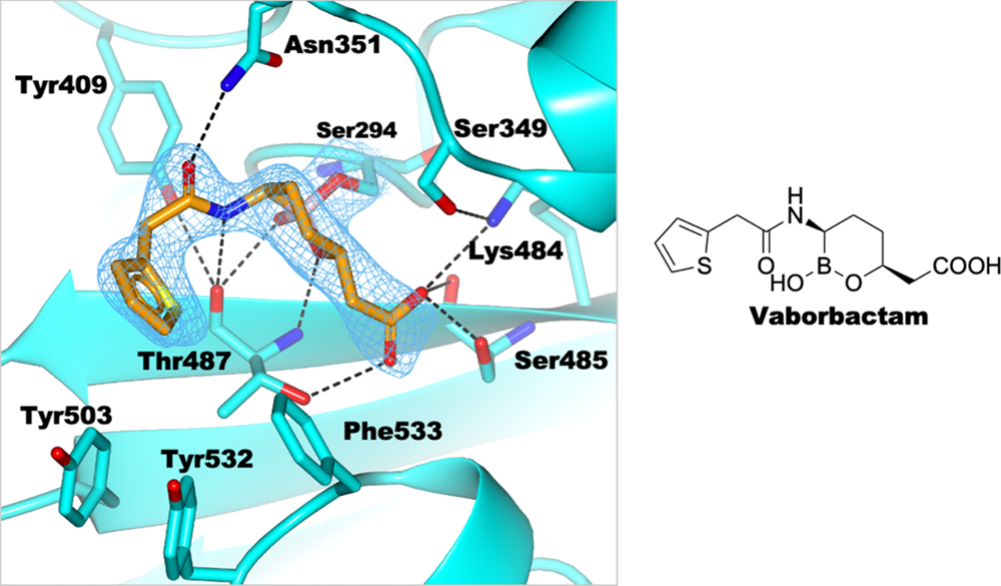
Binding of vaborbactam to PaPBP3. Hydrogen bonds: black dashed lines. An unbiased omit Fo-Fc map is shown (light blue mesh) of the ligand and covalently attached residue side chains (contoured at 1 σ), as calculated by “comit” in the ccp4 suite.^45^ PDB code 6AUH.

Vaborbactam, an approved monocyclic SBL inhibitor^17^ without reported antimicrobial activity, was included in our studies to investigate its potential interactions with PaPBP3 compared to the other BCIs. Our PaPBP3/vaborbactam structure shows a monocovalent reaction, as observed with SBLs^16^ (Figure 8). The structures of vaborbactam and amoxicillin in complex with PaPBP3 superimpose well, including with respect to the oxyanion hole, the C-3 carboxylate, their amide bonds, and in the position of their aromatic rings (Figure S16). Similar to amoxicillin, vaborbactam is positioned to form hydrogen bonds with residues including with Asn351, Tyr409, Thr487, and Ser485 as well as well to engage with the hydrophobic wall (residues Tyr503, Tyr532, and Phe533). Despite these favorable interactions, vaborbactam binds poorly to PaPBP3 in solution (Table 1), consistent with its lack of antimicrobial activity.

## Conclusions

The multiple PBP3 structures reported here with three different classes of boron-based compounds, that is, boronic acids, and predominantly mono- (vaborbactam) and bicyclic (benzoxaboroles) boronates, reveal three distinct binding modes. Vaborbactam forms a covalent bond reacting with the catalytic serine, whereas the benzoxaboroles form dicovalent complexes (additionally reacting with Ser349), and the boronic acids form tricovalent complexes (additionally reacting with Lys484). While these binding modes are precedented with other nucleophilic serine enzymes,^27,70,71^ the generality of the reactions is notable, in particular for the tricovalent reactions observed for the boronic acids. Given the binding modes as reflected by crystallography (though not as yet validated in solution) and that SBL/MBL inhibition by boron-containing compounds has been demonstrated, it is perhaps surprising that the compounds described here are not more potent PaPBP3 inhibitors though affinities for boronates against essential PBPs have typically been poor.^22−25^ Recent patent reports concerning compounds with a bicyclic boronate (taniborbactam-like) scaffold show promising activity against PBPs,^32,33^ but a clear explanation for the differential potencies of boronate inhibition of BLs versus PBPs has yet to be established.^8^ Our combined results imply that the rigid nature of the di- and tricovalent complexes formed may require rather precise derivatization of the boron scaffold to obtain potent inhibition. Consistent with this, our attempt to combine the benzoxaborole PBP3 structures with those of β-lactams (e.g., piperacillin) did not give potent inhibitors. An interesting observation from the crystallography was of the influence of the boron inhibitors on the conformation of the β5-α11 and α10-β3 loops. The results presented here and elsewhere suggest that conformational changes during active-site ligand binding are important for inhibition and (likely) also induced fit substrate binding. The “morphing” ability of boron-containing ligands, combined with high-throughput crystallography to reveal otherwise latent structural changes, is notable. However, it is presently difficult to predict how specific active-site interactions may influence conformational changes for the active site. Indeed, the results suggest that the lack of potency of the PBP inhibition of compounds described here and elsewhere may relate to non-optimal interactions with mobile regions in and around the PBP active site. Thus, from a medicinal chemistry perspective, it may be most efficient to employ a combination of structure-guided (core scaffold) and empirical (side chain) approaches.

Insights from our PaPBP3/benzoxaborole structures combined with reported structures for piperacillin-, aztreonam- and ceftazidime-reacted complexes provide a strong knowledge base that will enable further explorations of benzoxaboroles as non-β-lactam-based PBP inhibitors. The PaPBP3 structures with **14** and **15** are interesting as they indicate vectors to target key PBP active-site interaction hotspots for improving affinity. If benzoxaborole PaPBP3 potency can be improved, there is patent literature precedent that boron-based PBP inhibitors can achieve MICs of 8–16 μg/mL in CTX-M-15-expressing *E. coli* strains.^32,33^ Given the pressing need for novel antibiotics against resistant Gram-negative bacteria, further exploration of benzoxaboroles and related PBP inhibitors is a promising approach to new antibiotics targeting PBPs.

## Experimental Section

### Protein Synthesis and Crystallography

Genes encoding for recombinant PBP3s from *P. aeruginosa*, *E.coli*, *A. baumannii*, and *H. influenzae* were expressed as soluble fragments to produce proteins with PBP residues 50–579, 60–588, 64–609, and 80–610, respectively, which lack the *N*-terminal transmembrane helix anchor. A soluble construct expressing only the *C*-terminal transpeptidation domain of *N. gonorrhoeae* PBP2 (residues 237–581) (a class B PBP with close analogy to the PaPBP3 protein, with different nomenclatures for historical reasons)^55,56^ from the clinical mutation-mediated penicillin-resistant *N. gonorrhoeae* (FA6140) was used. All proteins were produced and purified as described using reverse nickel affinity chromatography utilizing the HRV 3C protease to cleave the N-terminal His6 expression tags.^51^

*Pa*PBP3 crystals were obtained at 294 K using the hanging drop vapor diffusion method by mixing equal volumes of 10 mg/mL protein with the following precipitant solutions: 25% (w/v) polyethylene glycol 3 350, 1% (w/v) protamine sulfate, and 0.1 M Bis-Tris propane at pH 6 or 8.^66^ Crystals were cryoprotected with 20% (v/v) glycerol prior to flash-freezing in liquid nitrogen. Inhibitor–protein complexes were obtained by soaking crystals overnight with a 250 mM solution of the requisite compound. Diffraction data were collected on Diamond beamlines I03, I04, and I04-1. All data were processed using autoPROC and STARANISO^72^ due to significant anisotropy of the data, with the exception of the complexes of PaPBP3 with **7** and **15**, which were isotropic and which were processed with autoPROC.^73^ Structures were phased by *Phaser_MR*(74) using a high-resolution structure of PaPBP3 (PDB: 6HZR).^51^ Manual building and ligand fitting were performed with COOT.^75^ Refinement was carried out primarily using REFMAC5^76^ within the CCP4 suite^45^ as well as in phenix.refine.^77^ Structures were validated with MolProbity.^78^ Figures of structures were prepared using PyMOL (The PyMOL Molecular Graphics System, Schrödinger, LLC) or CCP4mg.^79^

### BOCILLIN FL Assays

Unless otherwise stated, assays were run in triplicate using a 60 nM purified protein (see above), 30 nM BOCILLIN FL in pH 7 100 mM sodium phosphate buffer, with 0.01% Triton to reduce promiscuous ligand binding^23^ and reduce binding of the protein to the plate. Assays were run in triplicate in a volume of 50 μL, in black, flat-bottom, 384-well microplates (Grenier Bio-One, Austria) at 30 °C. The change in FA was measured using a ClarioStar plate reader (BMG Labtech) with polarized filters at excitation: 482–16, emission: F: 530–4, and calculated using MARS software v3.32 (BMG Labtech) using the equation
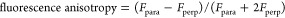
where *F*_para_ is the fluorescence intensity parallel to the excitation plane and *F*_perp_ is fluorescence intensity perpendicular to the excitation plane.

Residual activities were determined by pre-incubating the test compound (1024 μM) and protein for 1 h at 30 °C before the reaction was initiated by the addition of BOCILLIN FL. The change in FA after 30 min was compared to the uninhibited control to determine the residual activity. In order to calculate *K*_i_, the compound (at 11 concentrations: 1–1024 μM) and BOCILLIN FL were mixed and the reaction was initiated by the addition of PBP. Also included in the model were progress curves of nine concentrations of PBP (40–110 nM). Inhibition plots with meropenem and ceftazidime were done to ensure the correct parameters for BOCILLIN FL binding were used. Progress curves were analyzed with KinTek, USA, as described;^52,69^ reported errors represent the standard error. The acylation of a PBP by BOCILLIN FL (Figure S1) can be described by a simple one-step model.^52^ However, unlike the previous studies, we found it was necessary to include a term (*k*_2_) to account for the deacylation of BOCILLIN FL to release a hydrolyzed product (Figure S1). The fluorescence intensity was constant throughout the reaction. Inhibitor binding was modeled as a reversible reaction to form the enzyme–inhibitor complex (Figure S1). *K*_i_ was calculated as the ratio of the off-rate *k*_off_ to the on-rate *k*_on_ (Figure S1). Inhibitor binding fittings are shown in Figure S2.

### S2d Assays

Residual activities were measured by the ability of a potential inhibitor to hinder hydrolysis of the substrate analogue S2d (2-((benzoyl-d-alanyl)thio)acetic acid) turnover by PaPBP3 as described for other PBPs.^4,24,53,54^ Assays were conducted in 50 μL in a 384-well, clear-bottom, black-walled microplate (Greiner Bio-One). PaPBP3 (400 nM) was incubated with 2 mM of each compound for 1 h at 30 °C in 100 mM sodium phosphate, pH 7, supplemented with 0.01% (v/v) triton. A solution of 5,5′-dithiobis-(2-nitrobenzoic acid) and S2d diluted in the same buffer (to give a final concentration in the assay of 1 mM for both reagents) was added to each well to initiate the reaction. The final concentration of protein was 200 nM and the final inhibitor concentration was 1 mM. A ClarioStar plate reader (BMG Labtech) was used to follow the reaction by observing the change in absorbance at 412 nm at 30 °C. The same assay was conducted in the absence of the inhibitor and additionally with an excess of aztreonam (1 mM), which completely inhibits PaPBP3; the results of this were used as a control to determine the rate of spontaneous S2d hydrolysis in the absence of an enzyme. The initial rate of S2d turnover was calculated using GraphPad prism (Prism 8 for macOS, GraphPad Software LLC) and the standard error calculated from three independent technical replicates. The rate of non-enzymatic S2d hydrolysis was subtracted from each rate to find the corrected rate. For each compound, the ratio of the corrected rate to the untreated control corrected rate was expressed as a percentage to give the residual activity.
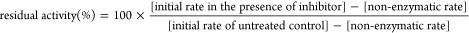


Methods for conducting the nitrocefin assay and microbiology are provided in the Supporting Information.

### Interference Considerations

The activity of all the compounds (**1**–**15**) belonging to the same class were analyzed by two orthogonal methods (FA and absorbance) with good correlation between the results of both assays. Triton (0.01% v/v) was added to the buffer for both assays, which should reduce interference by aggregators. Moreover, a nitrocefin dilution assay indicates that the interaction of **12** with PaPBP3 is reversible and well behaved (Figure S3). The weak interaction of **12** with closely related PBPs from other bacteria indicates that the reaction is selective (Table 2). Lastly, for boronic acids (PaPBP3/**1** and PaPBP3/**2**, Figure 2) and benzoxaboroles (PaPBP3 in complex with **3**, **4**, **7**, **12**, **13**, **14**, and **15**, Figure S8), multiple crystal structures demonstrate a conserved binding mode, specifically engaging active-site residues. The interaction is consistent with examples from the literature of nucleophilic residue engagement by boron-based compounds (Figure S17).

**1**–**15** were also screened *in silico* (http://zinc15.docking.org/patterns/home) for predicted PAINS and aggregator functional groups, but none were identified. During the design of the compounds (**1**–**15**), we aimed to obtain polar compounds to limit the chance of aggregation and avoided functional groups which can cause redox activity, fluorescence, protein reactivity, singlet-oxygen quenching, and so forth. The purity of each compound was >95%, minimizing the presence of impurities.

### Synthetic Chemistry

All reagents were from Sigma-Aldrich, Fisher Scientific, Combi-Blocks, Enamine, or Fluorochem and were used without further purification. **1** ((3-(1*H*-tetrazol-5-yl)phenyl)boronic acid) and **3** (1-hydroxy-1,3-dihydrobenzo[*c*][1,2]oxa-borole-6-carboxylic acid) were from Combi-Blocks, Inc. **2** ((5-methyl-1*H*-indazol-6-yl)boronic acid)) and **4** (4-(1-hydroxy-1,3-dihydrobenzo[*c*][1,2]oxaborole-6-carbonyl)-1,3,3-trimethylpiperazin-2-one) were from Enamine. These were used without further purification in the X-ray fragment screen. **3** was purchased from Combi-Blocks, Inc.; **12** and **13** were purchased from Wuxi Apptec; vaborbactam was purchased from MedChemExpress. 2-((Benzoyl-d-alanyl)thio)acetic acid (**S2d**) was synthesized as reported; the spectroscopic data were consistent with the ones previously reported.^80^

Solvents were used as received. Flash column chromatography was performed using a Teledyne ISCO flash purification system using a Silicycle SiliaSep C18 cartridge. Purity of all final derivatives for biological testing was confirmed to be >95% as determined using an Agilent ultra-performance liquid chromatograph–mass spectrometer (Agilent Technologies 6150 quadrupole, ES ionization) coupled with an Agilent Technologies 1290 Infinity II series UPLC system Agilent 1290 series high-performance LC (HPLC) at two wavelengths of 254 and 280 nm using the following conditions: Kinetex 1.7 μm Evo C18 100A, LC column 50 × 2.1 mm, solvent A of 0.1% (v/v) (formic acid) water, and solvent B of 0.1% (v/v) (formic acid) in acetonitrile. ^1^H and ^13^C nuclear magnetic resonance (NMR) spectra were recorded using a Varian Mercury 300 MHz spectrometer or a Bruker AVIII 600 MHz instrument. Deuterated solvents were used as supplied. Chemical shifts (δ), referenced using residual solvent peaks, are reported in parts per million downfield from residual solvent peak as an internal standard. Multiplicity is given as s (singlet), d (doublet), t (triplet), q (quartet), m (multiplet), br (broad), or a combination of these. Coupling constants, *J*, are reported in hertz (Hz) to the nearest 0.5 Hz. High-resolution mass spectra were recorded using a Bruker MicroTOF instrument with an electrospray ionization source and time of flight (TOF) analyzer. The parent ion is quoted with the indicated ion: [M – H]^−^ or [M + Na]^+^.

### General Protocol **1**: Amide Coupling

To a solution of the appropriate carboxylic acid (1 equiv) in *N*,*N*-DMF (2 mL) was added 1,1′-carbonyldiimidazole (CDI) (2 equiv). The reaction was stirred for 5 min at room temperature; the appropriate amine (1, 1.2, or 1.5 equiv) was then added, and the reaction was stirred for 4–16 h at 40 °C. The solvent was removed *in vacuo*, and the crude product was purified using a Teledyne ISCO CombiFlash chromatography system eluting with a reverse phase solvent gradient of MeOH in 0.1% (v/v) CH_3_CO_2_H/water and a C18 column. The product-containing fractions were then combined, and the organic solvent was removed *in vacuo*. When amide coupling yielded a target intermediate compound (e.g., methyl esters of general structure **B1**, Scheme 1), it was used in the next step without further purification, else lyophilization was used to afford the desired products as solids (i.e., for **5**–**10** and **14**).

### 1-Hydroxy-*N*-[2-(methylamino)-2-oxo-ethyl]-3*H*-2,1-benzoxaborole-6-carboxamide (**5**)

General Protocol 1 was followed using the following quantities of reagents: **3** (100 mg, 0.56 mmol, 1 equiv); *N,N*-DMF (2 mL); 1,1-carbonyldiimidazole (182 mg, 1.12 mmol, 2 equiv); 2-amino-*N*-methylacetamide HCl (104 mg, 0.84 mmol, 1.5 equiv). Product: crystalline solid (47 mg, 32%). Purity: >96% (by HPLC). ^1^H NMR (600 MHz, DMSO-*d*_6_): δ 9.55 (br s, 1H, O*H*), 8.71 (t, *J* = 6.0 Hz, 1H, N*H*), 8.18 (s, 1H, Ar–*H*), 7.93 (dd, *J* = 8.0, 2.0 Hz, 1H, Ar–*H*), 7.85 (q, *J* = 5.0 Hz, 1H, N*H*CH_3_), 7.49 (d, *J* = 8.0 Hz, 1H, Ar–*H*), 5.02 (s, 2H, −C*H*_2_OB), 3.85 (C*H*_2_, obscured by the solvent peak), 2.59 (d, *J* = 5.0 Hz, 3H, CH_3_); ^13^C NMR (151 MHz, DMSO-*d*_6_): δ 170.3, 167.8, 157.6, 133.2, 130.3, 130.1, 121.9, 70.4,43.1, 26.1; LCMS (ESI^+^, *m/z*), 249 [M + H]^+^; HRMS (ESI–TOF) calcd for C_11_H_13_N_2_O_4_^10^B; [M – H]^−^, 247.0896; found, 247.0895.

### *N*-[(1*R*)-2-Amino-1-benzyl-2-oxo-ethyl]-1-hydroxy-3*H*-2,1-benzoxaborole-6-carboxamide (**6**)

General Protocol 1 was followed using the following quantities of reagents: **3** (100 mg, 0.56 mmol, 1 equiv); *N,N*-DMF (2 mL); 1,1-carbonyldiimidazole (182 mg, 1.12 mmol, 2 equiv); (*R*)-2-amino-3-phenylpropanamide HCl (169 mg, 0.84 mmol, 1.5 equiv). Product: crystalline solid (80 mg, 43%). Purity: >98% (by HPLC). ^1^H NMR (600 MHz, DMSO-*d*_6_): δ 9.31 (br s, 1H, O*H*), 8.45 (d, *J* = 8.5 Hz, 1H, N*H*), 8.18–8.15 (m, 1H, Ar–*H*), 7.89 (dd, *J* = 8.0, 2.0 Hz, 1H, Ar–*H*), 7.59–7.52 (m, 1H, Ar–*H*), 7.46 (d, *J* = 8.0 Hz, 1H, Ar–*H*), 7.33–7.30 (m, 2H, N*H*_2_), 7.24 (t, *J* = 7.5 Hz, 2H, Ar–*H*), 7.20–7.13 (m, 1H, Ar–*H*), 7.12–7.08 (m, 1H, Ar–*H*), 5.02 (s, 2H, −C*H*_2_OB), 4.66 (ddd, *J* = 10.5, 8.5, 4.0 Hz, 1H, −C*H*NH), 3.12 (dd, *J* = 14.0, 4.0 Hz, 1H, −C*H*_2_Ph), 2.99 (dd, *J* = 14.0, 4.0 Hz, 1H, −C*H*_2_Ph); ^13^C NMR (151 MHz, DMSO-*d*_6_): δ 173.8, 166.9, 157.3, 139.0, 133.5, 130.3, 130.2, 129.6, 128.5, 126.7, 121.6, 70.4, 55.2, 37.7; LCMS (ESI^+^, *m/z*), 325 [M + H]^+^; HRMS (ESI–TOF) calculated for C_17_H_17_N_2_O_4_^10^B [M + Na]^+^, 347.1174; found, 347.1176.

### Methyl (2*R*)-2-[(1-hydroxy-3*H*-2,1-benzoxaborole-6-carbonyl)amino]-2-phenyl-Acetate (**7**)

General Protocol 1 was followed using the following quantities of reagents: **3** (100 mg, 0.56 mmol, 1 equiv); *N,N*-DMF (2 mL); 1,1-carbonyldiimidazole (182 mg, 1.12 mmol, 2 equiv); methyl (2*R*)-2-amino-2-phenyl-acetate (139 mg, 0.84 mmol, 1.5 equiv). Product: crystalline solid (38 mg, 20%). Purity: >96% (by HPLC). ^1^H NMR (600 MHz, DMSO-*d*_6_): δ 9.32 (br s, 1H, O*H*), 9.20 (d, *J* = 7.0 Hz, 1H, N*H*), 8.26 (t, *J* = 1.0 Hz, 1H, Ar–*H*), 7.99 (dd, *J* = 8.0, 2.0 Hz, 1H, Ar–*H*), 7.52–7.45 (m, 3H, Ar–*H*), 7.43–7.31 (m, 3H, Ar–*H*), 5.68 (d, *J* = 7.0 Hz, 1H, −NHC*H*), 5.04 (s, 2H, −C*H*_2_OB), 3.66 (s, 3H, −OC*H*_3_); ^13^C NMR (151 MHz, DMSO-*d*_6_): δ 171.6, 167.4, 157.7, 137.6, 136.7, 133.0, 130.7, 129.3, 128.7, 127.6, 70.4, 57.4, 52.8; LCMS (ESI^+^, *m/z*), 326 [M + H]^+^; HRMS (ESI–TOF) calculated for C_17_H_16_N_1_O_5_^10^B [M + Na]^+^, 348.1014; found, 348.1016.

### *N,N*-Dibenzyl-1-hydroxy-3*H*-2,1-benzoxaborole-6-carboxamide (**14**)

General Protocol 1 was followed using the following quantities of reagents: **3** (100 mg, 0.56 mmol, 1 equiv); *N,N*-DMF (2 mL); 1,1-carbonyldiimidazole (182 mg, 1.12 mmol, 2 equiv); dibenzylamine (133 mg, 0.67 mmol, 1.2 equiv). Product: crystalline solid (20 mg, 10%). Purity: >99% (by HPLC).^1^H NMR (300 MHz, DMSO-*d*_6_): δ 9.26 (br s, 1H, O*H*), 7.85 (s, 1H, Ar–*H*), 7.61–7.52 (m, 1H, Ar–*H*), 7.47 (d, *J* = 8.0 Hz, 1H, Ar–*H*), 7.44–7.08 (m, 10H, Ar–*H*), 5.02 (s, 2H, −C*H*_2_OB), 4.75–4.24 (m, 4H, 2× −C*H*_2_Ph). ^13^C NMR (151 MHz, DMSO-*d*_6_): δ 171.6, 167.4, 157.7, 136.7, 133.0, 130.7, 130.6, 129.3, 129.0, 128.7, 121.7, 70.4, 57.4, 52.7; LCMS (ESI^+^, *m/z*), 358 [M + H]^+^; HRMS (ESI–TOF) calcd for C_22_H_20_N_1_O_3_^10^B [M + Na]^+^, 380.1429; found, 380.1429.

### General Protocol 2: Synthesis of **8**, **9**, and **10** (Scheme 1A)

Step (i): General Protocol **1** was followed to afford an appropriate methyl ester intermediate, **B1**, which was then directly subjected to saponification. Step (ii): To a solution of **B1** (1 equiv) in 1,4-dioxane/water (3:1; 10 mL) was added lithium hydroxide monohydrate (either 4 or 6 equiv.) in one portion. The reaction mixture was then stirred for 1 h at 40 °C before the volatiles were removed *in vacuo*. The residue thus obtained was then lyophilized to afford corresponding free carboxylic acids (confirmed by LC–MS analysis) as solids. Step (iii): Crude carboxylic acids were immediately coupled with selected piperazin-2-one derivatives using conditions outlined in *General Protocol* 1, giving target benzoxaboroles as solids.

### 1-Hydroxy-*N*-[2-oxo-2-(3-oxopiperazin-1-yl)ethyl]-3*H*-2,1-benzoxaborole-6-carboxamide (**8**)

General Protocol 2 was followed with the following quantities of reagents: step (i): **3** (250 mg, 1.40 mmol, 1 equiv); *N,N*-DMF (2 mL); 1,1-carbonyldiimidazole (455 mg, 2.8 mmol, 2 equiv.) methyl glycinate HCl (133 mg, 0.67 mmol, 1.2 equiv). Step (ii): methyl 2-[(1-hydroxy-3*H*-2,1-benzoxaborole-6-carbonyl)amino]acetate (349 mg, 1.40 mmol, 1 equiv); 1,4-dioxane/water (3:1; 10 mL); lithium hydroxide monohydrate (235 mg, 5.61 mmol, 4 equiv). Step (iii): 2-[(1-hydroxy-3*H*-2,1-benzoxaborole-6-carbonyl)amino]acetic acid (50 mg, 0.21 mmol, 1 equiv); *N,N*-DMF (2 mL); 1,1-carbonyldiimidazole (69 mg, 0.69 mmol, 2 equiv); piperazin-2-one (32 mg, 0.32 mmol, 1.5 equiv). Product: crystalline solid [14 mg, 4% (over three steps)]. Purity: >97% (by HPLC). ^1^H NMR (600 MHz, DMSO-*d*_6_): δ 9.37 (br s, 1H, O*H*), 8.63–8.56 (m, 1H, N*H*), 8.33–8.20 (m, 1H, Ar–*H*), 7.96 (dd, *J* = 8.0, 2.0 Hz, 1H, Ar–*H*), 7.51 (d, *J* = 8.0 Hz, 1H, Ar–*H*), 5.05 (s, 2H, −C*H*_2_OB), 4.21–4.10 (m, 3H, C*H*_2_), 3.96 (s, 1H, C*H*_2_), 3.72–3.61 (m, 2H, C*H*_2_), 3.32–3.18 (m, 2H, C*H*_2_); ^13^C NMR (151 MHz, DMSO-*d*_6_): δ 172.1, 155.5, 135.4, 129.2, 129.2, 129.2, 129.1, 129.1, 127.9, 127.6, 127.5, 122.1, 70.4, 52.1; LCMS (ESI^+^, *m/z*), 318 [M + H]^+^; HRMS (ESI–TOF) calcd for C_14_H_16_N_3_O_5_^10^B [M + Na]^+^, 340.1076; found, 340.1076.

### (*R*)-1-Hydroxy-*N*-(1-(4-methyl-3-oxopiperazin-1-yl)-1-oxo-3-phenylpropan-2-yl)-1,3-dihydrobenzo[*c*][1,2]oxaborole-6-carboxamide (**9**)

*General Protocol* 2 was followed using the following quantities of reagents: Step (i): **3** (100 mg, 0.56 mmol, 1 equiv); DMF (2 mL); 1,1-carbonyldiimidazole (182 mg, 1.12 mmol, 2 equiv); methyl d-phenylalaninate hydrochloride (121 mg, 0.56 mmol, 1 equiv). Step (ii): methyl(1-hydroxy-1,3-dihydrobenzo[*c*][1,2]oxaborole-6-carbonyl)-d-phenylalanine (187 mg, 0.55 mmol, 1 equiv); 1,4-dioxane/water (3:1; 10 mL); lithium hydroxide monohydrate (139 mg, 3.31 mmol, 6 equiv). Step (iii): (1-hydroxy-1,3-dihydrobenzo[*c*][1,2]oxaborole-6-carbonyl)-d-phenylalanine (30 mg, 0.09 mmol, 1 equiv); *N,N*-DMF (2 mL); 1,1-carbonyldiimidazole (30 mg, 0.18 mmol, 2 equiv); 1-methylpiperazin-2-one (16 mg, 0.14 mmol, 1.5 equiv). Product: crystalline solid [16 mg, 8% (over 3 steps)]. Purity: >97% (by HPLC). ^1^H NMR (300 MHz, DMSO-*d*_6_): δ 8.85 (br s, 1H, O*H*), 8.24–8.16 (m, 1H, N*H*), 7.98–7.86 (m, 1H, Ar–*H*), 7.48 (d, *J* = 8.0 Hz, 1H, Ar–*H*), 7.36–7.14 (m, 6H, Ar–*H*), 5.20–4.95 (m, 2H, −C*H*_2_OB), 4.20–3.93 (m, 2H), 3.34–2.96 (m, 5H), 2.80 (s, 3H); LCMS (ESI^+^, *m/z*), 422 [M + H]^+^.

### 1-Hydroxy-*N*-[2-(4-methyl-3-oxo-piperazin-1-yl)-2-oxo-ethyl]-3*H*-2,1-benzoxaborole-6-carboxamide (**10**)

*General Protocol* 2 was followed with the following quantities of reagents: Step (i): 3 (250 mg, 1.40 mmol, 1 equiv); *N,N*-DMF (2 mL); 1,1-carbonyldiimidazole (455 mg, 2.8 mmol, 2 equiv.) methyl glycinate HCl (133 mg, 0.67 mmol, 1.2 equiv). Step (ii): methyl 2-[(1-hydroxy-3*H*-2,1-benzoxaborole-6-carbonyl)amino]acetate (349 mg, 1.40 mmol, 1 equiv); 1,4-dioxane/water (3:1; 10 mL); lithium hydroxide monohydrate (235 mg, 5.61 mmol, 4 equiv). Step (iii): 2-[(1-hydroxy-3*H*-2,1-benzoxaborole-6-carbonyl)amino]acetic acid (47 mg, 0.2 mmol, 1 equiv); *N*,*N*-DMF (2 mL); 1,1-carbonyldiimidazole (65 mg, 0.4 mmol, 2 equiv); 1-methylpiperazin-2-one (34 mg, 0.30 mmol, 1.5 equiv). Product: crystalline solid [17 mg, 5% (over 3 steps)]. Purity: >96% (by HPLC).^1^H NMR (300 MHz, DMSO-*d*_6_): δ 8.58 (br s, 1H, O*H*), 8.26 (s, 1H, Ar–*H*), 7.97 (d, *J* = 6.0 Hz, 1H, Ar–*H*), 7.51 (d, *J* = 8.0 Hz, 1H, Ar–*H*), 5.05 (s, 2H, C*H*_2_), 4.24–4.07 (m, 3H, −C*H*_2_OB and C*H*_2_), 4.05–3.94 (m, 2H, C*H*_2_), 3.48–3.30 (m, 2H, C*H*_2_), 2.89 (s, 3H, −NC*H*_3_); ^13^C NMR (151 MHz, DMSO-*d*_6_): δ167.6, 167.3, 165.2, 157.5, 133.4, 130.2, 130.0, 121.8, 70.3, 48.0, 47.5, 46.2, 41.2, 33.9; LCMS (ESI^+^, *m/z*), 332 [M + H]^+^.

### Synthesis of **11** (Scheme 1B)

#### Step (i)

To a solution of (**11a**) (*tert*-butoxycarbonyl)-d-phenylalanine) (300 mg, 1.13 mmol) in *N,N*-DMF (3 mL) was added 1,1-carbonyldiimidazole (367 mg, 2.26 mmol); the reaction was stirred for 5 min at room temperature. To the reaction mixture was then added piperazin-2-one (170 mg, 1.70 mmol) and the resultant solution was stirred for 4 h at 40 °C. Ethyl acetate (10 mL) and water (10 mL) were added. The layers were separated. The aqueous layer was extracted with ethyl acetate (3 × 20 mL). Combined organic layers were dried (MgSO_4_), filtered, and concentrated *in vacuo* to afford **11b** (*tert*-butyl *N*-[(1*R*)-1-benzyl-2-oxo-2-(3-oxopiperazin-1-yl)ethyl]carbamate) as a yellow oil (350 mg, 88%), which was used in the next step without further purification.

#### Step (ii)

To a stirred solution of (**11b**) (350 mg, 1.01 mmol, 1 equiv) in CH_2_Cl_2_ (10 mL) was added HCl (4 M in 1,4-dioxane, 0.76 mL, 3.02 mmol, 3 equiv) at room temperature under nitrogen. The reaction mixture was stirred overnight to afford an insoluble precipitate. The precipitate, **11c**, was washed three times with CH_2_Cl_2_ and then filtered, dried in air, and used in the next step without further purification (54 mg, 19%).

#### Step (iii)

*General Protocol* 1 was followed using the following quantities of reagents: **3** (54 mg, 0.3 mmol, 1 equiv); *N,N*-DMF (3 mL); 1,1-carbonyldiimidazole (98 mg, 0.600 mmol, 2 equiv); **11c** (103 mg, 0.360 mmol, 1.2 equiv). Product (**11**): white powder (20 mg, 16%). Purity: >99% (by HPLC). ^1^H NMR (300 MHz, methanol-*d*_4_): δ 8.31 (br s, 1H, O*H*), 8.14 (dd, *J* = 8.0, 2.0 Hz, 2H, Ar–*H*), 7.93 (d, *J* = 8.0 Hz, 1H, Ar–*H*), 7.56–7.45 (m, 2H, Ar–*H*), 7.36–7.22 (m, 3H, Ar–*H*), 5.15 (m, 5H, 2× C*H*_2_ and C*H*), 4.33–3.96 (m, 2H, C*H*_2_), 3.88–3.40 (m, 3H, C*H*_2_), 3.28–3.06 (m, 3H, C*H*_2_); ^13^C NMR (151 MHz, DMSO-*d*_6_): δ 170.5, 167.3, 167.3, 157.8, 137.7, 132.1, 131.9, 130.3, 129.6, 128.7, 127.1, 122.5, 70.4, 51.5, 46.1, 42.3, 40.4, 37.5; LCMS (ESI^+^, *m/z*), 408 [M + H]^+^; HRMS (ESI–TOF) calcd for C_21_H_22_N_3_O_5_^10^B [M + Na]^+^, 430.1543; found, 430.1546.

### Synthesis of **15** (Scheme 1C)

Intermediates **15a** and **15b** were prepared as previously described^81,82^

#### Synthesis of (4-(benzyloxy)-2-formylphenyl)boronic Acid Pinacol Ester (**15c**)

Bis(pinacolato)diboron (0.618 g, 2.43 mmol) (**15b**), 5-(benzyloxy)-2-bromobenzaldehyde (0.500 g, 1.71 mmol), and potassium acetate (0.478 g, 4.82 mmol) were dissolved in 1,4-dioxane (15 mL); the suspension was then degassed with nitrogen, after which PdCl_2_(dppf) (0.119 g, 0.16 mmol) was added. The reaction mixture was refluxed for 12 h before it was allowed to cool to room temperature. Solids were then removed by filtration through Celite washing with EtOAc (100 mL). The filtrate was concentrated *in vacuo* and the residue obtained was purified by Teledyne ISCO CombiFlash automated chromatography (gradient of EtOAc in hexane, 0–30%) to give a white solid (490 mg, 86%). Purity: >99% (by HPLC). ^1^H NMR (300 MHz, chloroform-*d*): δ 10.69 (s, 1H), 7.90 (d, *J* = 8.5 Hz, 1H), 7.62 (d, *J* = 2.5 Hz, 1H), 7.44 (tt, *J* = 4.0, 2.0 Hz, 2H), 7.41 – 7.37 (m, 3H), 7.22 (dd, *J* = 8.5, 2.5 Hz, 1H), 5.17 (s, 2H), 1.39 (s, 13H), 1.29 (s, 12H). LCMS (ESI^+^, *m*/*z*): mass not observed.

#### Synthesis of Ethyl 2-(5-(Benzyloxy)-1-hydroxy-1,3-dihydrobenzo[*c*][1,2]oxaborol-3-yl)acetate (**15d**)

To a stirred solution of ethyl acetate (0.32 g, 3.63 mmol, 1.5 equiv) in dry tetrahydrofuran (THF) was added lithium diisopropylamide (LDA, 3.55 mL, 3.63 mmol, 1.5 equiv, 1 M solution in THF) dropwise at −78 °C. The mixture was stirred for 30 min at −78 °C before (4-(benzyloxy)-2-formylphenyl)boronic acid pinacol ester (0.8 g, 2.36 mmol, 1 equiv) was added. Next, the reaction mixture was stirred for 2 h at −20 °C before it was quenched by addition of a saturated solution of NH_4_Cl (10 mL). EtOAc (10 mL) was added and the layers were separated. The aqueous layer was extracted with EtOAc (3 × 10 mL). The combined organic extracts were washed with brine, dried (MgSO_4_), and then concentrated *in vacuo*. The residue obtained was then purified by Teledyne ISCO CombiFlash automated chromatography (gradient of EtOAc in hexane in 0–30%) to give the desired product as a white solid (160 mg, 57%). Purity >99% (by HPLC). ^1^H NMR (300 MHz, methanol-*d*_4_): δ 7.62–7.53 (m, 1H), 7.51–7.30 (m, 5H), 7.04–6.97 (m, 2H), 5.53 (dd, *J* = 8.5, 4.5 Hz, 1H), 5.14 (s, 2H), 4.18 (qd, *J* = 7.0, 1.5 Hz, 2H), 2.95 (dd, *J* = 15.5, 4.5 Hz, 1H), 2.55 (dd, *J* = 15.5, 8.5 Hz, 1H), 1.26 (t, *J* = 7.0 Hz, 3H). LCMS (ESI^+^, *m*/*z*), 327 [M + H]^+^.

#### Synthesis of 2-(5-(Benzyloxy)-1-hydroxy-1,3-dihydrobenzo[*c*][1,2]oxaborol-3-yl)acetic Acid (**15**)

To a stirred solution of ethyl 2-(5-(benzyloxy)-1-hydroxy-1,3-dihydrobenzo[c][1,2]oxaborol-3-yl)acetate (0.3 g, 0.92 mmol, 1 equiv) in THF/H_2_O (10 mL, 1:1) was added LiOH·H_2_O (77 mg, 1.84 mmol, 2 equiv); the mixture was then stirred for 2 h at room temperature. Water (20 mL) and ethyl acetate (20 mL) were then added and the layers were separated. The aqueous layer was acidified to pH 2–3 and then extracted with ethyl acetate (3 × 20 mL). The layers were separated and the combined organic extracts were washed with brine, dried (MgSO_4_), and then concentrated *in vacuo*. On standing at room temperature overnight, the product solidified. Further washing with diethyl ether gave the purified product as a white solid (210 mg, 78%). Purity >97% (by HPLC).^1^H NMR (300 MHz, methanol-*d*_4_): δ 7.58 (d, *J* = 8.0 Hz, 1H), 7.49–7.32 (m, 5H), 7.07–6.98 (m, 2H), 5.54 (dd, *J* = 8.5, 4.5 Hz, 1H), 5.14 (s, 2H), 2.89 (dd, *J* = 15.5, 4.5 Hz, 1H), 2.51 (dd, *J* = 15.5, 8.5 Hz, 1H). ^13^C NMR (151 MHz, DMSO-*d*_6_): δ 172.4, 161.4, 158.8, 137.3, 132.2, 128.9, 128.4, 128.4, 115.5, 107.6, 77.3, 69.8, 42.3. LCMS (ESI^+^, *m*/*z*), 299 [M + H]^+^.

## Supporting Information Available

PDB model of a docked PaPBP3:**11** complex (PDB)
Methods for antimicrobial and nitrocefin assays and details of the fragment selection process; computational chemistry protocols; model and inhibition curves for the BOCILLIN FL and nitrocefin assays; microbiological data; crystallography statistics and crystallization pHs for each model; additional crystal structure views; examples of boron-based inhibition of other targets via reaction with nucleophilic residues; and HPLC traces for lead compounds (PDF)
Molecular formula strings (CSV)

## Supplementary Material

jm1c00717_si_001.pdb

## Notes

PaPBP3 in complex with **1** (7ATM), **2** (7ATO), **3** (7ATW), **4** (7ATX), **7** (7AU0), **12** (7AU1), **13** (7AU8), **14** (7AU9), **15** (7AUB) and vaborbactam (7AUH). The authors will release the atomic coordinates and experimental data upon article publication.

## Abbreviations

BL: β-lactamase
CDI: 1,1′-carbonyldiimidazole
DBO: diazabicyclooctane
FA: fluorescence anisotropy
MBL: metallo-β-lactamase
ND: not determined
PaPBP3: *Paeruginosa* PBP3
PBP: penicillin-binding protein
PDB: protein data bank
S2d: 2-((benzoyl-d-alanyl)thio)acetic acid
SBL: serine β-lactamase
