# Supplementary material for: High-Throughput Crystallography Reveals Boron-Containing Inhibitors of a Penicillin-Binding Protein with Di- and Tricovalent Binding Modes
Source: J Med Chem. 2021 Jul 31;64(15):11379–94. doi: 10.1021/acs.jmedchem.1c00717 (PMC9282634; doi:10.1021/acs.jmedchem.1c00717)
Supplement: Supplementary file 2 — jm1c00717_si_002.pdf [file jm1c00717_si_002.pdf]

Supporting Information

High-Throughput Crystallography Reveals Boron Containing Inhibitors of a Penicillin Binding Protein with Di- and Tri-covalent Binding Modes

Hector Newman, [ab] Alen Krajnc,[c] Dom Bellini,[a] Charles J. Eyermann,[d*] Grant A. Boyle[d], Neil G. Paterson,[b] Katherine E. McAuley,[b] Robert Lesniak,[c] Mukesh Gangar,[d] Frank von Delft,[befg] Jürgen Brem,[c] Kelly Chibale,[dh] Christopher J. Schofield[c*] and Christopher G. Dowson[a*]

[a] School of Life Sciences, University of Warwick, Coventry, CV4 7AL, United Kingdom.

[b] Diamond Light Source Ltd., Harwell Science and Innovation Campus, Didcot, OX11 0DE, United Kingdom.

[c] Department of Chemistry and the Ineos Oxford Institute of Antimicrobial Research, Chemistry Research Laboratory, 12 Mansfield Road, Oxford, OX1 3TA, United Kingdom.

[d] Drug Discovery and Development Centre (H3D), University of Cape Town, Rondebosch 7701, South Africa.

[e] Structural Genomics Consortium (SGC), University of Oxford, Oxford, United Kingdom.

[f] Department of Biochemistry, University of Johannesburg, Auckland Park 2006, South Africa.

[g] Research Complex at Harwell, Harwell Science and Innovation Campus, Didcot, OX11 0FA, United Kingdom.

[h] South African Medical Research Council Drug Discovery and Development Research Unit, Department of Chemistry and Institute of Infectious Disease and Molecular Medicine, University of Cape Town, Rondebosch 7701, South Africa.

* Corresponding Authors: CJE, eyermanncj@gmail.com; CJS, christopher.schofield@chem.ox.ac.uk; CGD, C.G.Dowson@warwick .ac.uk

Supplementary Methods **S3**

Kinetics **S6**

Microbiology **S8**

Crystallography **S9**

HPLC traces **S28**

References **S33**

Supplementary Methods

Antimicrobial Assays Minimum inhibitory concentration (MIC) were determined for CLSI reference strains of the following Gram negative organisms: *E. coli* (NCTC 25922)*, P. aeruginosa (*PAO1 and a permeabilized strain which was a kind gift from HI Zgurskaya and colleagues[1]), *H. Influenza* (ATCC 49766)*, A. baumannii* (ATCC 19606) and *N. gonorrhoeae* (ATCC 49226) by the broth microdilution method using a control antibiotic. CLSI procedures were strictly adhered to throughout, with the exception of total volume, which was reduced to minimize compound consumption. The *E. coli* and *P. aeruginosa* strains were tested in a 10 µL final volume in cation-adjusted Mueller Hinton broth on the lids of inverted 96 well Costar microplates (Corning, USA) and incubated at 37 °C for 18 hours in a humidor (~98% humidity) to reduce evaporation. *H. Influenzae*, *A. baumannii* and *N. gonorrhoeae* were tested in 50 µL of cation-adjusted Mueller Hinton broth supplemented with 5% lysed blood in 96-well Costar microplates (Corning, USA) and incubated at 37 °C for 18 hours in approx. 5% CO_2. Growth was determined by visual inspection of the plate after the incubation period.

Synergy studies were conducted using the checkerboard titration method [2] with *E. coli* and *P. aeruginosa* in a total volume of 10 µL cation adjusted Mueller Hinton broth, piperacillin at concentrations from 32-0.25 µg/mL and benzoxaborole at 64-1 µg/mL, diluted in perpendicular directions across the plate. Growth was determined by visual inspection of the plate after 18 hours at 37 °C at 95 % humidity.

Nitrocefin Assays [3–5] Nitrocefin was used to determine the effect of a two-fold dilution on the pIC_{50} of **12** (Figure S3). Nitrocefin (150 µM, Abcam UK) turnover by *P. aeruginosa* PBP3 (PaPBP3) (238 nM) was monitored at 482 nM using a ClarioStar plate reader (BMG Labtech) in clear bottom 384 well plates (Greiner Bio-One,) The volume was initially 40 µL, made up in 50 mM bis-tris propane, pH 8.5, containing 1 % (v/v) Triton X-100 and 20 mM MgCl. Assays (30 °C) were initiated by enzyme addition and allowed to react for 120 s; an additional 40 µL of buffer was

then added (final volume: 80 µL). The plate was shaken for a total of 140 s at 500 rpm and another 120 s of reading were taken. Rates of these linear curves were calculated using the plate-reader's software MARS (BMG Labtech) and compared to the untreated control to determine relative rates.

Fragment Selection Data pipelining software KNIME v3.5.3 [6] was used to select fragments from the "Serine Focused Covalent Fragments" compound set from Enamine. Diversity was assessed using the "Diversity Picker" module by RDKit, which implements the MaxMin algorithm.[7,8] Further compounds were added manually.

Computational Chemistry The benzoxaborole di-covalent binding mode of compound **3** in complex with PaPBP3 was used as a template for modelling benzoxaborole design ideas. Given the β5-α11 loop was not defined in the PaPBP3: **3** structure (PDB: 7ATX), presumably due to its inability to engage the residues on the β5-α11 loop, a model of **3** bound to the PaPBP3 structure as observed in the piperacillin-reacted PaPBP3 structure (PDB: 6R3X [9]) was developed. All modelling studies were performed using the Schrodinger Suite of programs (Schrodinger LLC, New York, NY). Benzoxaborole design ideas which incorporate the key binding interactions between reacted piperacillin and PaPB3 were built by modifying compound **3** using Schrodinger's 3D Builder tool. All designs, including compound **11**, were docked into PaPBP3 using the Glide SP software. Glide SP docking calculations were performed on a noncovalently bound ligand where the oxaborole portion of the benzoxaborole was deleted and the remaining phenyl group was constrained to the position observed in PaPBP3:**3**. Hydrogen-bond constraints to Tyr328, Asn351 and Arg489 were applied in the Glide SP docking. The Glide docking poses of the inhibitor were then reconstituted to a complete covalently bound benzoxaborole and the inhibitor and PaPBP3 residues within 6 Å of the inhibitor were then minimized using Prime. The only explicit water included in the minimization calculations was a crystallographically observed (in PaPBP3:**3**) water that hydrogen bonds to the backbone NHs of Ser294 and Thr487 (**w** in Figure 4b). The minimizations were performed using the OPLS2005

force field, the variable-dielectric generalized Born (VSGB) solvation model for water, a dielectric constant of 80, and 40 iterations of 200 steps each.

Kinetics

Bocillin

$$E + B \xrightarrow{k_1} EB \xrightarrow{k_2} E + P$$

Inhibitor

$$E + I \underset{k_{off}}{\overset{k_{on}}{\rightleftharpoons}} EI$$

$$K_i = k_{off} / k_{on}$$

Figure S1. Model used to determine K_i in Kintek Global Explorer.[10] **E, B, EB, P, I** and **EI** represent the PBP, BOCILLIN FL, enzyme-BOCILLIN FL acyl complex, hydrolyzed BOCILLIN FL, the inhibitor and the enzyme-inhibitor complex, respectively. k_1 models the acylation rate of BOCILLIN FL and k_2 the deacylation rate of BOCILLIN FL.[11] K_i is the ratio of the off- and on-rates (k_{off} and k_{on}, respectively) of the inhibitor binding to the PBP.

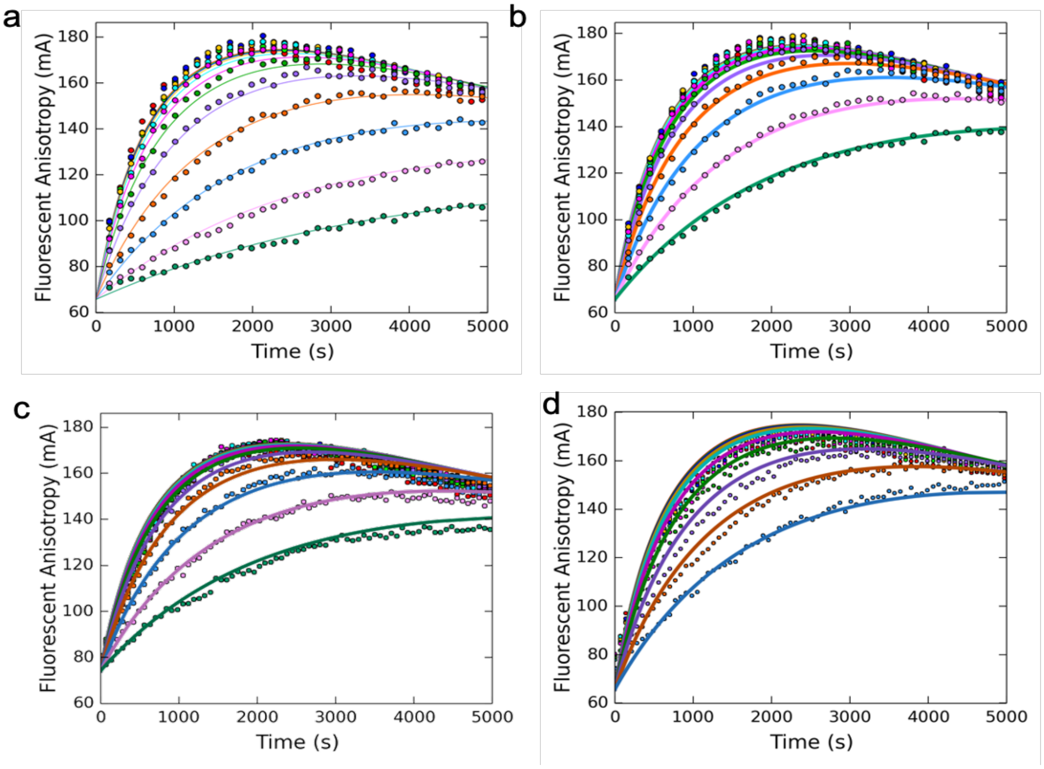

Figure S2. Inhibition curves fit to a reversible binding model (Figure S1). Datasets are shown for (a) **12**, (b) **13**, (c) **15** and (d) **Vaborbactam**. Kintek Global explorer was used to fit 11 concentrations of inhibitor from 1 mM to 1 μM and determine K_i values (Table 1).

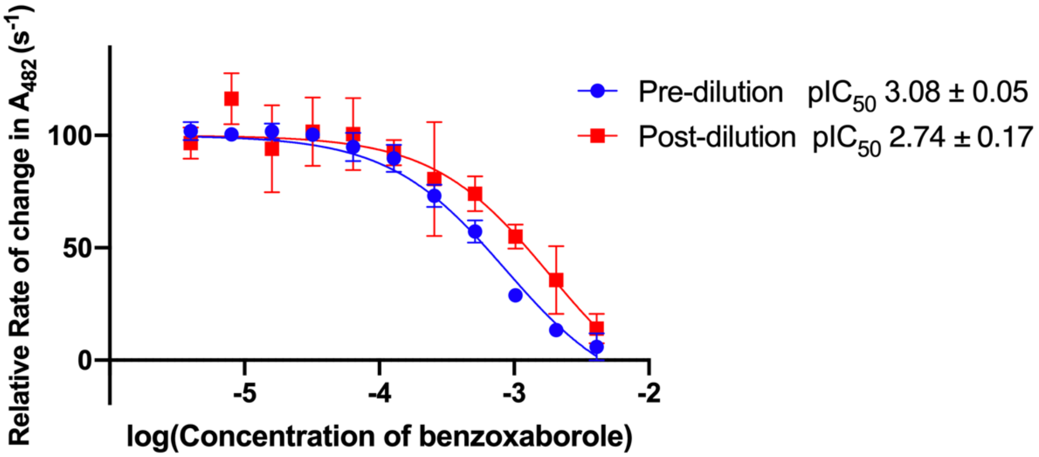

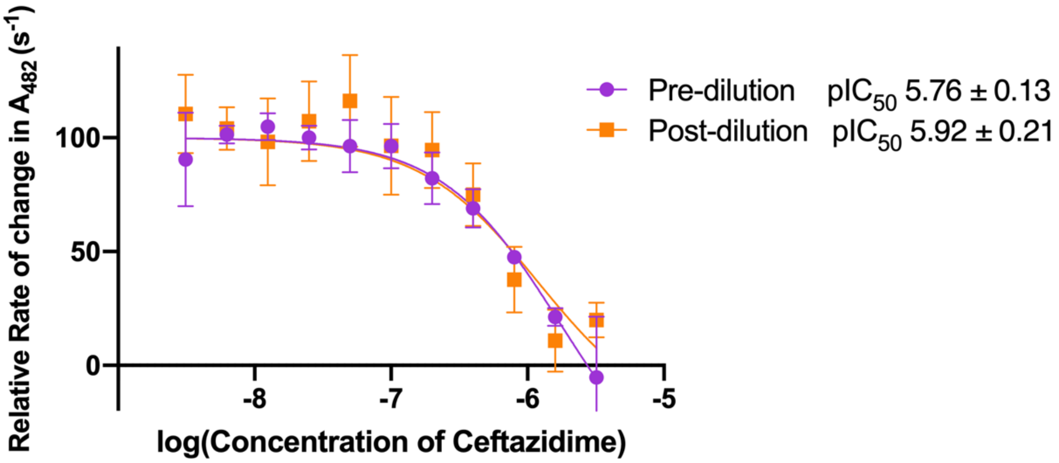

Figure S3. pIC_{50} s of benzoxaborole 12 and ceftazidime with PaPBP3 and nitrocefin before and after 2-fold dilution. The rate of nitrocefin turnover (measured at 482 nm) in the presence and absence of inhibitor was determined before and after a 2-fold dilution; rates were compared to the uninhibited control. Errors shown for each point are standard deviations from three repeats, errors on pIC_{50} values are standard errors of the mean of the pIC_{50} as determined by Prism 8 (Graphpad Software, LLC).

Microbiology

Table S1. Minimum inhibitory concentrations tested for a panel of Gram-negative bacteria.

	P. aeruginosa PAO1	*E. coli* NCTC25922	*P. aeruginosa* Permeabilized [a]	*N. gonorrhoeae* ATCC 49226	*A. baumanii* ATCC 19606	*H. influenzae* ATCC 49766	*P. aeruginosa* PAO1: piperacillin synergy	*E. coli* NCTC 25922: piperacillin synergy
**7**	>64 µg/mL	>64 µg/mL	>64 µg/mL	NT	NT	NT	NT	NT
**12**	>64 µg/mL	>64 µg/mL	>64 µg/mL	>64 µg/mL	>64 µg/mL	>64 µg/mL	No Effect	No Effect
**13**	>64 µg/mL	>64 µg/mL	>64 µg/mL	NT	NT	NT	NT	NT
**14**	>64 µg/mL	>64 µg/mL	>64 µg/mL	NT	NT	NT	NT	NT

[a]Permeabilized strain with the introduction of a FhuA pore and knock out of export pumps.[1]

Crystallography

Table S2. Crystallography Statistics

Dataset	PaPBP3:1 PDB Code: 7ATM	PaPBP3:2 PDB Code: 7ATO	PaPBP3:3 PDB Code: 7ATW	PaPBP3:4 PDB Code: 7ATX	PaPBP3:7 PDB Code: 7AU0
**Beamline**	DLS I03	DLS I03	DLS I03	DLS I03	DLS I04-1
**Wavelength**	0.97624	0.97934	0.97934	0.97934	0.91587
**Resolution range (Å)** [a]	1.58 –59.82 (1.58 -1.72)	1.59 - 61.11 (1.59 -1.71)	1.44 - 60.25 (1.44 - 1.59)	1.77 – 60.88 (1.77– 1.88)	2.17- 60.45 (2.17 - 2.21)
**Space group**	P 21 21 21	P 21 21 21	P 21 21 21	P 21 21 21	P 21 21 21
**Unit cell**	69.85 80.95 88.80 90 90 90	68.25 83.16 90.12 90 90 90	69.30 82.24 88.52 90 90 90	68.92, 83.03, 89.53 90 90 90	68.575 82.676 88.616 90 90 90
**Unique reflections**	53811	53748	67983	44742	27129
**Multiplicity**[a]	8.7 (8.2)	7.4 (7.4)	7.1 (6.0)	7.3 (7.6)	8.8 (8.6)
**Completeness (%)**[b]	95.3 (62.9)	95.5 (65.4)	95.7 (67.6)	95.5 (52.2)	99.9 (99.9)
**Mean I/sigI**[a]	15.8 (1.5)	15.4 (1.4)	18.0 (1.8)	16.4 (1.2)	8.2 (1.0)
**Rmeas**[a]	0.069 (1.51)	0.064 (1.426)	0.048 (0.952)	0.056 (1.683)	0.201 (2.816)
**CC½**[a]	1.0 (0.5)	1.0 (0.6)	1.0 (0.7)	1.0 (0.5)	1.0 (0.5)
**R-work**	0.1906	0.1498	0.1407	0.2088	0.1985
**R-free**	0.2246	0.2343	0.2010	0.2645	0.2619
**Number of non-hydrogen atoms**	3926	4133	4062	3982	3846
**macromolecules**	3669	3866	3826	3809	3685
**ligands**	22	21	30	21	23
**solvent**	235	246	206	152	138
**RMS(bonds)**	0.02	503	0.02	497	0.02
**RMS(angles)**	1.82	0.016	2	0.014	1.93
**Ramachandran outliers (%)**	0	1.98	0	1.86	0.42
**Rotamer outliers (%)**	1.03	0.2	0.74	0	2.34
**Average B-factor**	35.64	2.22	36.6	3.24	54.3
**macromolecules**	35.45	44.11	36.24	53.61	54.54
**ligands**	40.59	44.07	55.86	53.73	63.14
**solvent**	38.11	35.89	40.31	52.91	46.39

Dataset	PaPBP3:12 PDB Code: 7AU1	PaPBP3:13 PDB Code: 7AU8	PaPBP3:14 PDB Code: 7AU9	PaPBP3:15 PDB Code: 7AUB	PaPBP3: Vaborbactam PDB Code: 7AUH
**Beamline**	DLS I04-1	DLS I04-1	DLS I04-1	DLS I03	DLS I04
**Wavelength**	0.91587	0.91587	0.91587	0.97625	0.97950
**Resolution range (Å)** [a]	1.36 – 60.04 (1.36 - 1.49)	1.79 – 60.57 (1.79 – 1.97)	2.14 - 60.45 (2.14 – 2.34)	1.91 - 60.72 (1.91 – 1.94)	2.01 – 61.13 (2.01 – 2.22)
**Space group**	P 21 21 21	P 21 21 21	P 21 21 21	P 21 21 21	P 21 21 21
**Unit cell**	68.038 81.925 88.241 90 90 90	68.996 82.871 88.735 90 90 90	68.700 82.724 88.538 90 90 90	69.256 82.977 89.089 90 90 90	68.175 83.659 89.530 90 90 90
**Unique reflections**	69775	35335	21028	40602	25128
**Multiplicity**[a]	8.1 (5.7)	9.1 (12.0)	8.6 (7.8)	7.2 (6.4)	8.5 (6.7)
**Completeness (%)**[b]	91.7 (62.5)	94.9 (65.1)	94.1 (67.2)	99.7 (98.3)	94.0 (62.9)
**Mean I/sigI**[a]	15.1 (1.6)	16.7 (1.5)	12.9 (1.6)	16.7 (0.9)	15.5 (1.5)
**Rmeas**[a]	0.077 (0.935)	0.074 (1.613)	0.129 (1.404)	0.058 (1.801)	0.079 (1.127)
**CC½**[a]	1.0 (0.7)	1.0 (0.7)	1.0 (0.6)	1.0 (0.4)	1.0 (0.7)
**R-work**	0.1445	0.1969	0.1919	0.1985	0.2131
**R-free**	0.2033	0.2567	0.2743	0.2456	0.2820
**Number of non-hydrogen atoms**	4508	3999	3871	3829	4026
**macromolecules**	4009	3754	3739	3657	3863
**ligands**	91	27	32	21	26
**solvent**	408	218	100	151	137
**RMS(bonds)**	0.02	0.014	0.01	0.01	0.015
**RMS(angles)**	2.11	1.82	1.89	1.83	1.92
**Ramachandran outliers (%)**	0	0	0.83	0.21	0.4
**Rotamer outliers (%)**	1.16	2.8	3.84	1.83	2.48
**Average B-factor**	22.25	47.55	52.13	55.72	51.3
**macromolecules**	20.84	47.8	52.37	55.95	51.56
**ligands**	37	47.21	55.78	59.02	48.35
**solvent**	32.82	43.24	41.79	49.75	42.82

[a]Values for the highest resolution shell are in parentheses. [b]All data, except for PaPBP3:**7** and PaPBP3:**15**, were processed using STARANISO (Global Phasing) and the ellipsoidal completeness is given.[12,13] PaPBP3:**7** and PaPBP3:**15** were processed with autoPROC (Global Phasing)[14]; the value given is the spherical completeness.

Table S3. Crystallization pH and observed binding modes[a]

Structure	Core	Binding Mode	pH
PaPBP3:**1**	Phenylboronic acid	Tri-covalent	8
PaPBP3:**2**	Phenylboronic acid	Tri-covalent	8
PaPBP3:**3**	Benzoxaborole	Di-covalent	6
PaPBP3:**4**	Benzoxaborole	Di-covalent	8
PaPBP3:**7**	Benzoxaborole	Di-covalent	6
PaPBP3:**12**	Benzoxaborole with 3-carboxylic acid group	Di-covalent	6
PaPBP3:**13**	Benzoxaborole with 3-carboxylic acid group	Di-covalent	8
PaPBP3:**14**	Benzoxaborole	Di-covalent	6
PaPBP3:**15**	Benzoxaborole with 3-carboxylic acid group	Di-covalent	6
PaPBP3:**Vaborbactam**	Monocyclic, 6-membered boron-containing ring	Mono-covalent	8

[a]Ligands were soaked into crystals of PaPBP3 at pH 6 or pH 8; the pH that gave the best density for the ligand was selected for PDB deposition. Good models were fit to data collected at both pHs; note that for the available data, the pH does not appear to alter the observed binding mode.

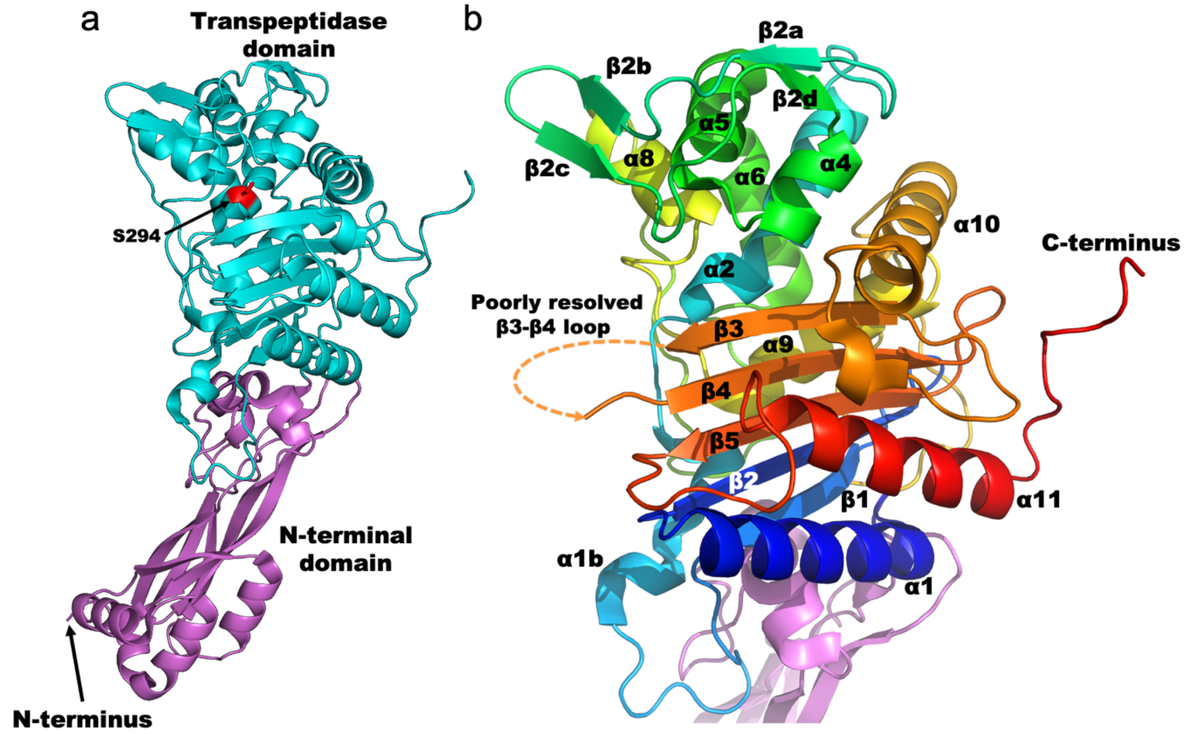

Figure S4. Views from a crystal structure of PaPBP3. (a) PaPBP3 (PDB: 6HZR)[9] has two domains: an N-terminal domain (pink) and a transpeptidase domain (cyan). The position of the active site catalytic serine is in red. The first 50 residues (a transmembrane domain) of the N-terminus were removed to enable solubilization; the N-terminus of the resultant protein is labeled.[9,15,16] (b) Secondary structure of the transpeptidase domain of PaPBP3, colored by rainbow (from dark blue at N-terminal end, to red at C terminal end). The N-terminal domain is in pink. Secondary structure assignment follows that of Pares *et al.*[17]

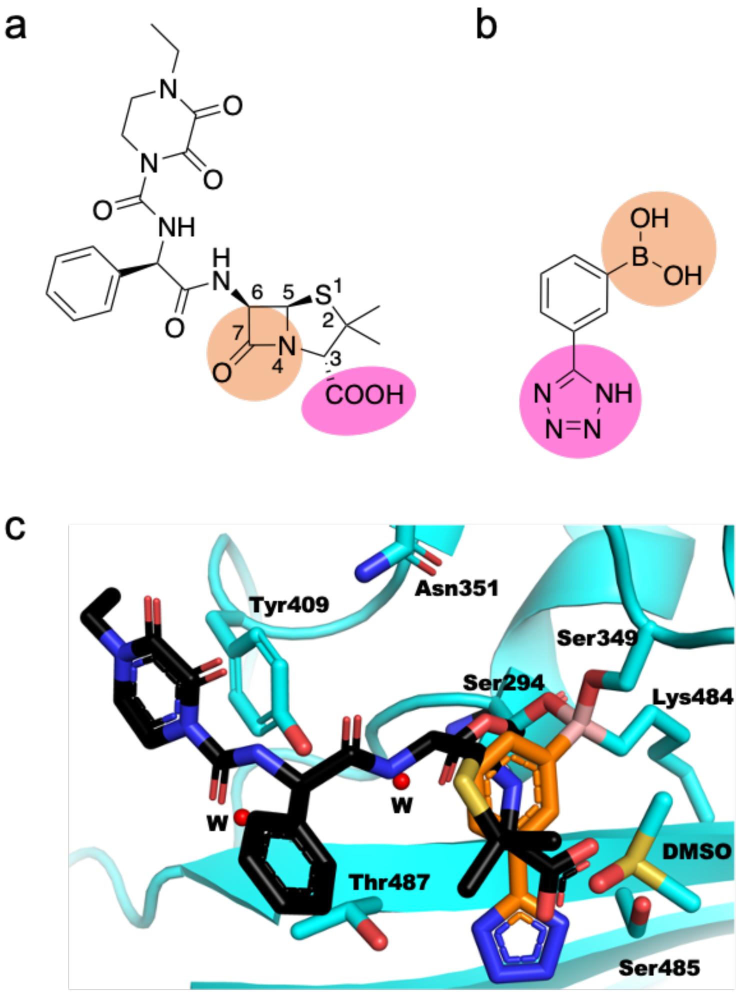

Figure S5. Comparison of binding modes of the piperacillin derived adduct and 1. Structures of (a) piperacillin and (b) **1** are shown, with their reactive groups highlighted in orange and the group engaging the acid binding pocket in pink. (c) View from a structure of PaPBP3:**1** (orange, PDB: 7ATM), with a view from the piperacillin-reacted structure overlaid (black, PDB: 6R3X).[9] A molecule of DMSO in the active site of PaPBP3:**1** is shown, as are selected water molecules (w).

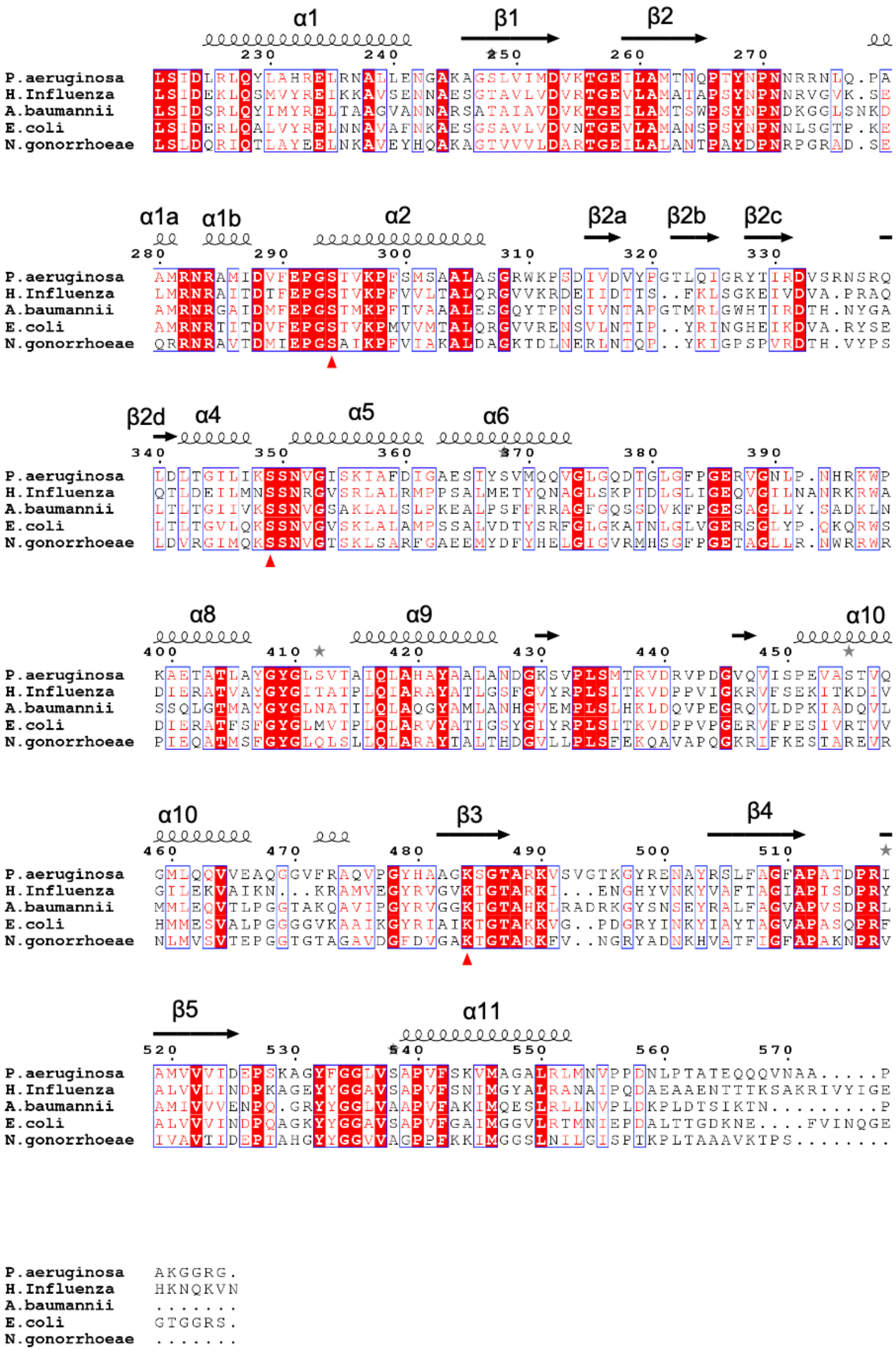

Figure S6. Comparisons of sequences of the transpeptidase domains of PBP3 from *P. aeruginosa*, *H. Influenzae, A. baumannii* and *E.coli*, and PBP2 from *N. gonorrhoeae*. Secondary structure elements are labeled, with the assignments following those of Pares *et al.*[4] The (potentially) boron-reacting residues Ser294, Ser349 and Lys484 (PaPBP3 numbering) are indicated with a red arrow. The alignment was done using ClustalOmega,[18] and the figure was generated using ESPript.[19]

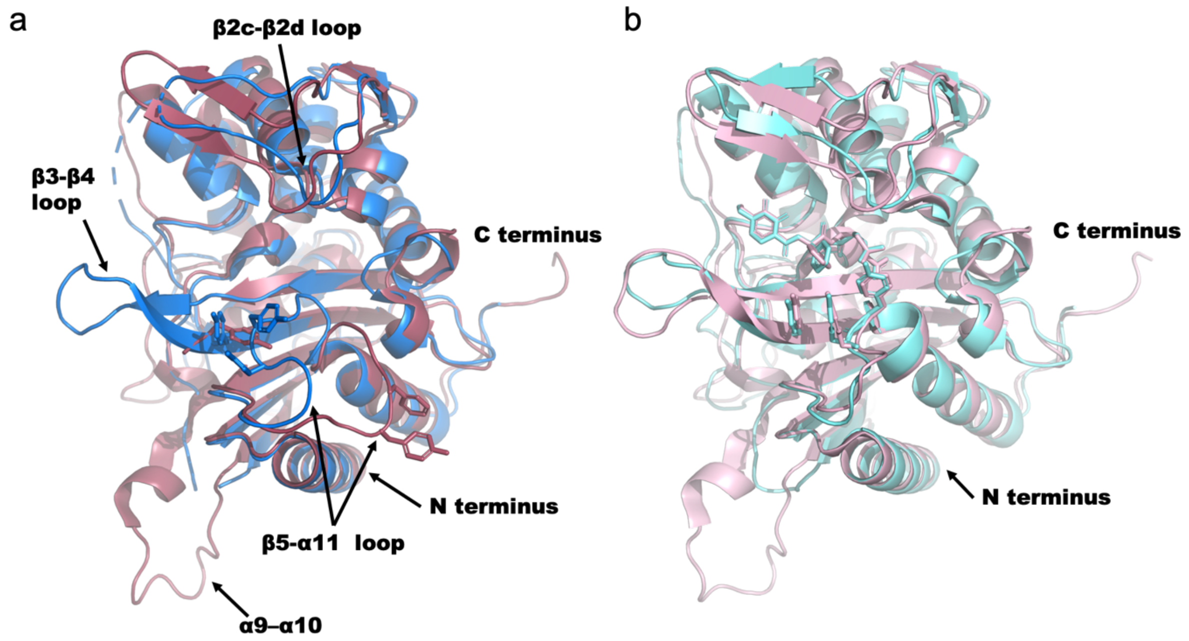

Figure S7. Comparisons of the transpeptidase domains of *P. aeruginosa* and *E. coli* PBP3. (a) Unligated structures of PaPBP3 (burgundy, PDB: 6R3X)[9] and *E. coli* PBP3 (EcPBP3) (a structure of the transpeptidase domain only, blue, PDB: 6HZQ).[9] (b) Piperacillin-reacted structures of PaPBP3 (pink, PDB: 6HZR)[9] and EcPBP3 (cyan, PDB: 6I1I)[9]. Substantial differences in the structures are seen for the β2c- β2d loop, β3-β4 loop and the β5-α11 loop (labeled). The β5-α11 loop is closer to the active site in the unligated EcPBP3, but is extended in unligated PaPBP3. In the piperacillin-reacted complex (b), both structures overlay well in this region. In the unligated PaPBP3 structure, the β3-β4 loop is too flexible to be observed crystallographically. The ligated PaPBP3 and EcPBP3 structures have slightly different conformations of the β2c- β2d loop. To aid crystallisation, the α9-α10 loop of EcPBP3 was replaced with a single glycine,[9] so this region does not appear in the apo or piperacillin-reacted EcPBP3 structures

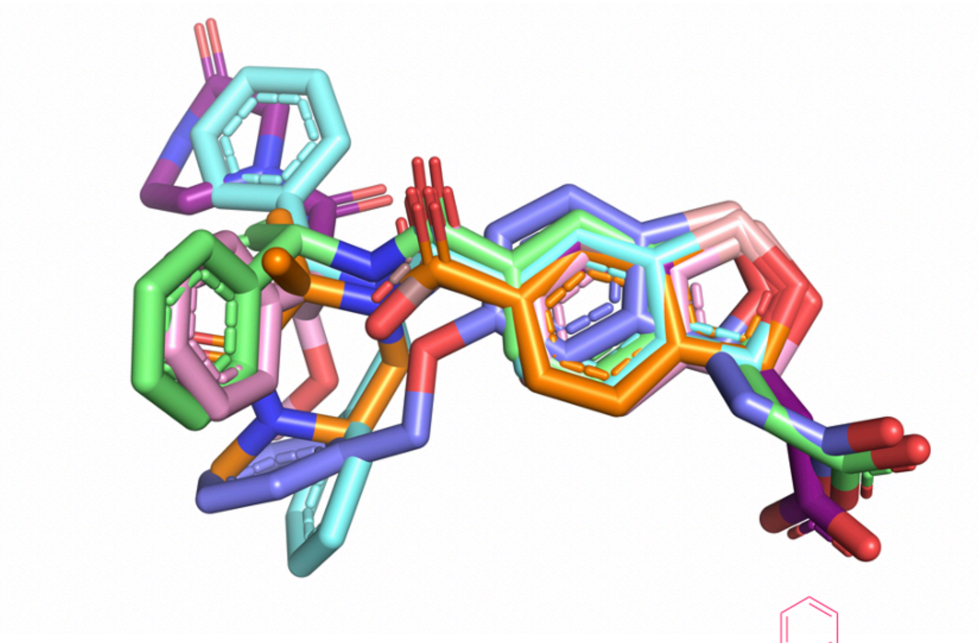

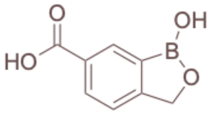
3

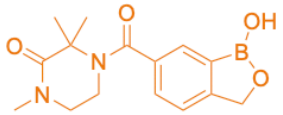
4

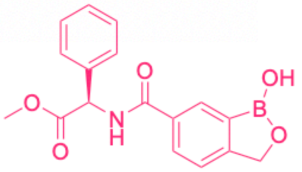
7

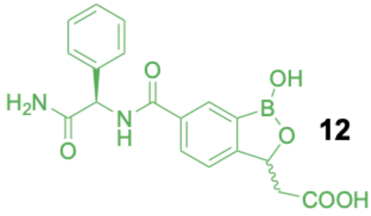
12

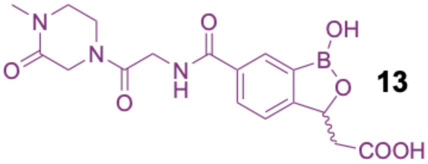
13

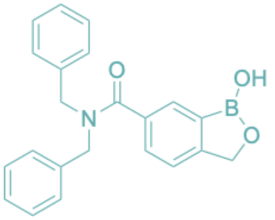
14

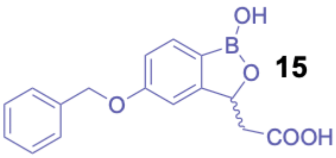
15

Figure S8. Overlay of benzoxaborole conformations as observed in complex with PaPBP3. Views of crystal structures of PaPBP3 with benzoxaborole compounds **3** (brown, PDB: 7ATW), **4** (orange, PDB: 7ATX), **7** (pink, PDB: 7AU0), **12** (green, PDB: 7AU1), **13** (purple, PDB: 7AU8), **14** (cyan, PDB: 7AU9), **15** (blue, PDB: 7AUB), reveal that they bind in a conserved mode. The protein structure is hidden for clarity. All compounds bind di-covalently: the tetrahedral boron reacts with the hydroxyls of Ser294 and Ser349 (not shown for clarity).

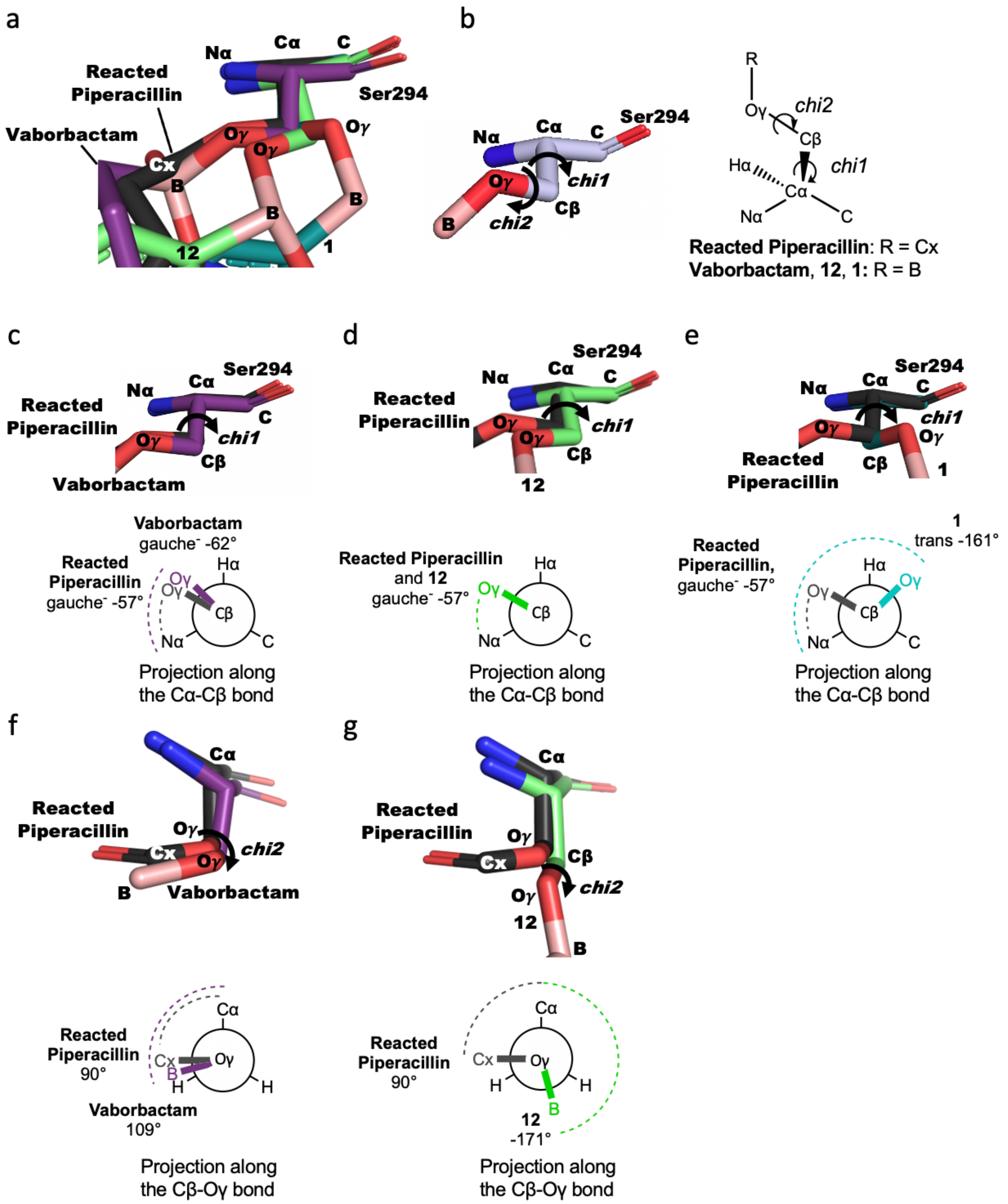

Figure S9. Crystal structure views and Newman projections of Ser294 reacted with boron-containing inhibitors (BCIs). (a) Enlarged view of Figure 6d. Superimposition of 3 views of the Ser294 alcohol bound to BCIs (**1, 12** and **Vaborbactam**; PDB: 7ATM, 7AU1 and 7AUH respectively) and reacted with piperacillin (PDB: 6R3X)[9]. Ser349 (which reacts with the boron of **12** and **1**) and Lys484 (which reacts with the boron of **1**) are not shown. The position of the boron in each of the views in Figure7d is associated with different Ser294 *chi1* and *chi2* angles; (b) Atoms and relevant angles of Ser294; (c-g) Views and Newman projections aligned along the Ser294 Cα-Cβ bond (c-e) and the Ser294 Cβ-Oγ bond (f and g) for each of **1, 12** and **Vaborbactam**) compared to the piperacillin-reacted structure (PDB: 6R3X)[9]. *Chi1* dihedral

angles are defined relative to the Ser294 Nα, *Chi2* dihedral angles are defined relative to the Ser294Cα. The PaPBP3:**Vaborbactam** structure (purple) and the piperacillin-reacted structure (black) both have *gauche*$^-$ Ser294 *chi1* angles (-62° and -57°, respectively) (c) and similar Ser294 *chi2* angles (109° and 90°, respectively) (f). The PaPBP3:**12** structure (green) has the same Ser294 *chi1* angle as the piperacillin-reacted structure (-57°) (d), but a nearly orthogonal Ser294 *chi2* angle (-171° and 90°, respectively) (g). The PaPBP3:**1** structure (teal) has a *chi1* angle that is *trans* to the Ser294 Nα, similar to the other reported tri-covalent structure of a boronate bound to a PBP (which has a Ser49 *chi1* angle of 179° (Figure S17)).[20] Other di-covalently binding benzoxaboroles (**3**, **4**, **7**, **9**, **13**, **14**, and **15**) have similar *chi1* and *chi2* angles to **12**; the tri-covalently binding **2** has similar Ser294 bond angles to **1**. In summary, mono-covalent structures (**Vaborbactam** and β-lactams) and di-covalent structures (benzoxaboroles) are differentiated by their Ser294 *chi2* angles, but have similar (gauche$^-$) Ser294 *chi1* angles. Tri-covalent structures (for phenyl boronic acids **1** and **2** and in previous observations [20]) have trans serine *chi1* angles.

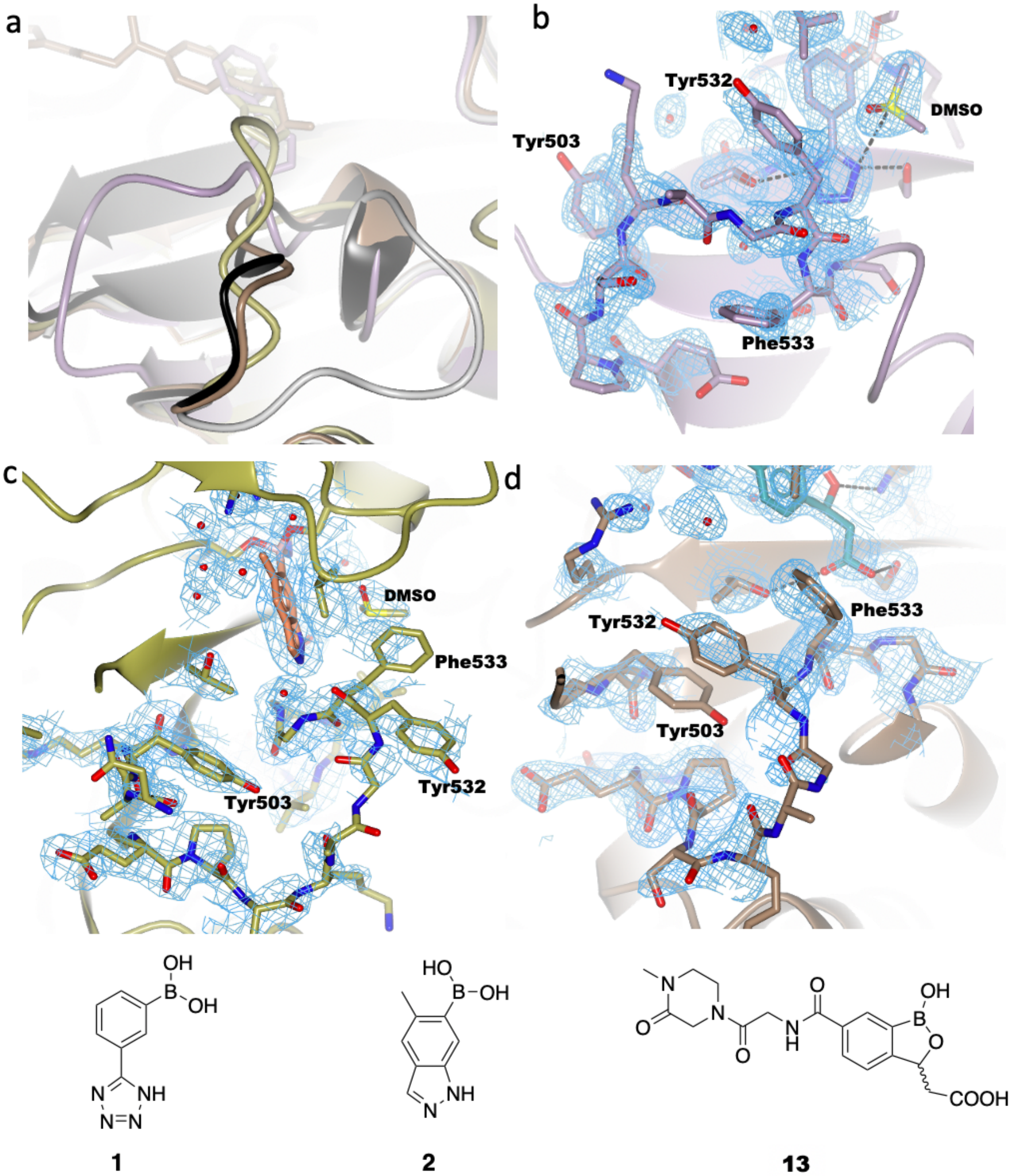

Figure S10. The PaPBP3 β5-α11 loop conformation is dynamic (a) Representation of the β5-α11 loop (residues 528-539) in structures with **1** (lilac, PDB: 7ATM), **2** (yellow, PDB: 7ATO), **13** (brown, PDB: 7AU8), and piperacillin (black, reacted piperacillin not shown for clarity PDB: 6R3X)[9] bound to PaPBP3, as well as the unligated-structure (white, PDB: 6HZR).[9] Views of the loop in structures of PaBP3 with: (b) **1**, (c) **2** and (d) **13**. Note that in (b) and (c), a DMSO molecule is present in the active site. For the PaPBP3:**13** complex, the main chain conformation is similar to that in the piperacillin-reacted PaPBP3, but residues Tyr503, Tyr532 and Phe333 π-stack in a different orientation. Electron densities (blue mesh) are unbiased composite omit maps contoured at 1 σ, produced using the 'comit' function in the CCP4 suite.[21]

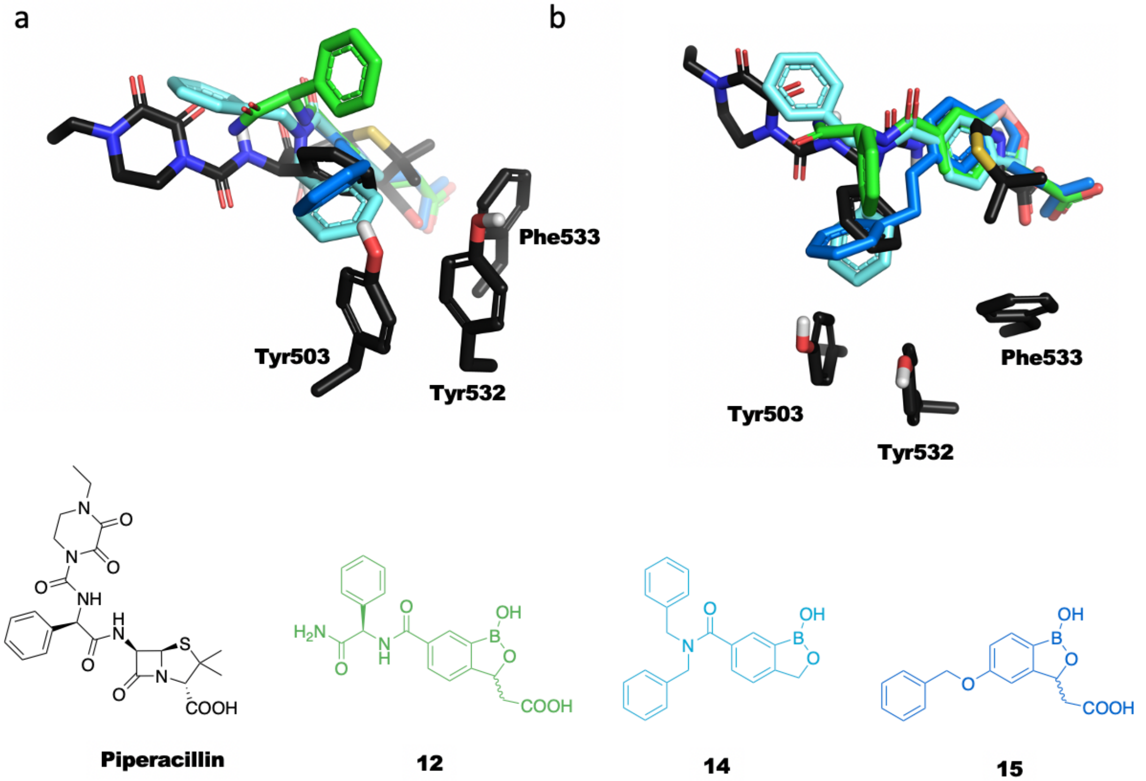

Figure S11. Phenyl groups of ligands designed to engage with the "hydrophobic wall" residues are not observed to do so. Crystallographically observed conformations of PaPBP3 complexed with **12** (green, PDB: 7AU1), **14** (cyan, PDB: 7AU9) and **15** (blue, PDB: 7AUB) and reacted piperacillin (black, PDB: 6R3X) are shown[9]. The reacted piperacillin phenyl ring is positioned to engage the hydrophobic wall (residues Tyr503, Tyr532 and Phe533, shown in black), but attempts to recreate these interactions with phenyl-substituted benzoxaboroles failed. In each case, the interaction with the wall was not formed. Side (a) and (b) 'top' view of the hydrophobic wall.

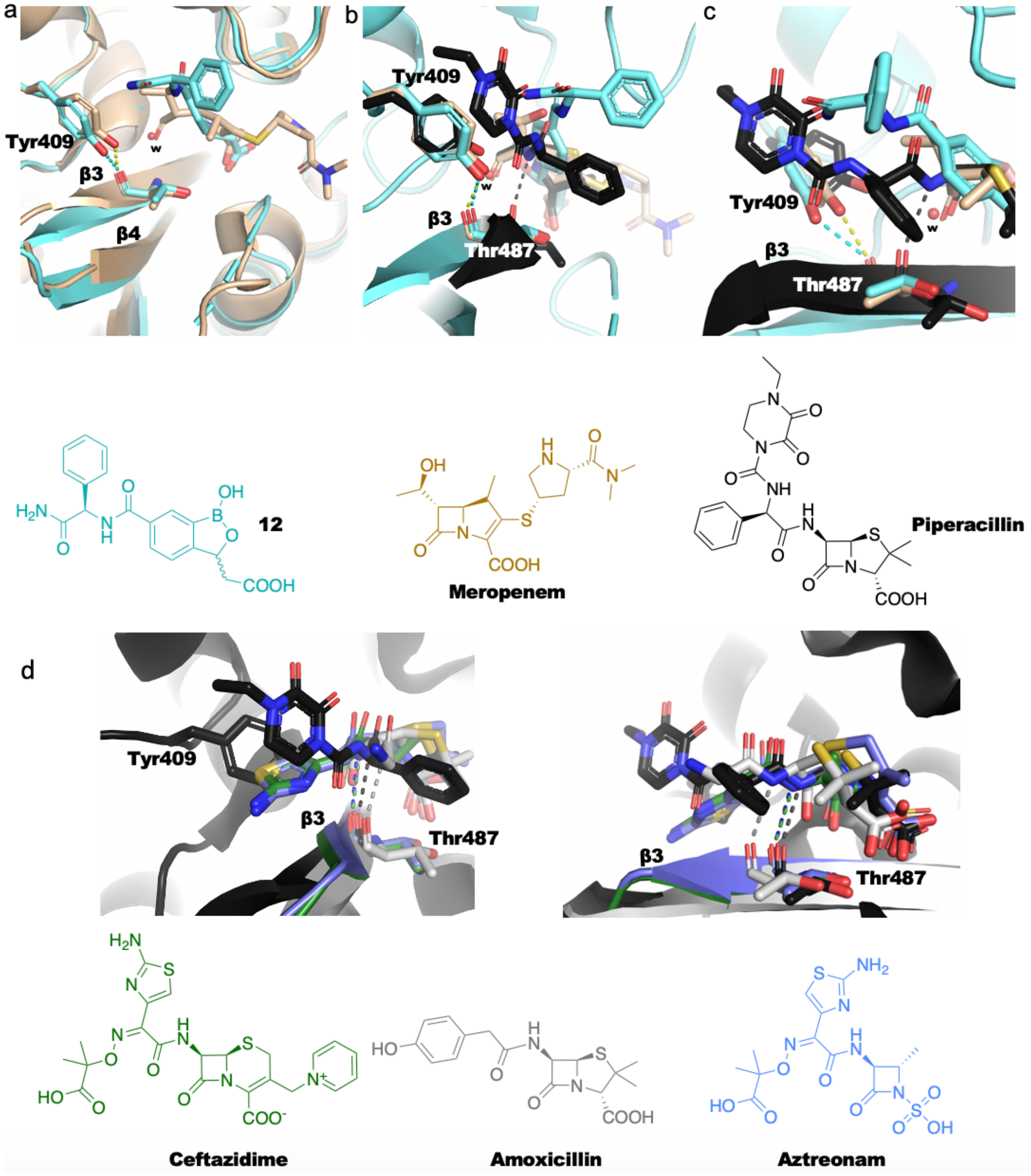

Figure S12. PaPBP3:meropenem and PaPBP3:12 structures reveal similar conformations for β3, which is different to the β3 conformation observed with other β-lactam complexes. (a) Active site view of the PaPBP3:meropenem complex (beige, PDB: 3PBR)[22] compared to PaPBP3:**12** (cyan, PDB: 7AU1). (b and c) Views of the β3 conformation in PaPBP3:**12** (cyan) compared to PaPBP3:meropenem (beige) and piperacillin-reacted complex (black, PDB: 6R3X)[9]. The hydrogen bond between Tyr409 and the backbone amine of Thr487 is shown in the respective color schemes. The hydrogen bond between Thr487 and the reacted piperacillin amine is in dashed black lines. The protein backbone shown is for the PaPBP3:**12** complex (cyan), with the β3 from PaPBP3 overlaid (black. (d) The β-lactam C-6 amide nitrogen:Thr487 hydrogen bond is shown for amoxicillin- (white, PDB: 6I1E)[9], piperacillin- (black, PDB: 6R3X)[9], ceftazidime- (green, PDB: 3PBO)[22] and aztreonam-reacted (blue, PDB: 3PBS)[22] PaPBP3 crystal structures; note the hydrogen bond (dashed lines) is similarly positioned in each view.

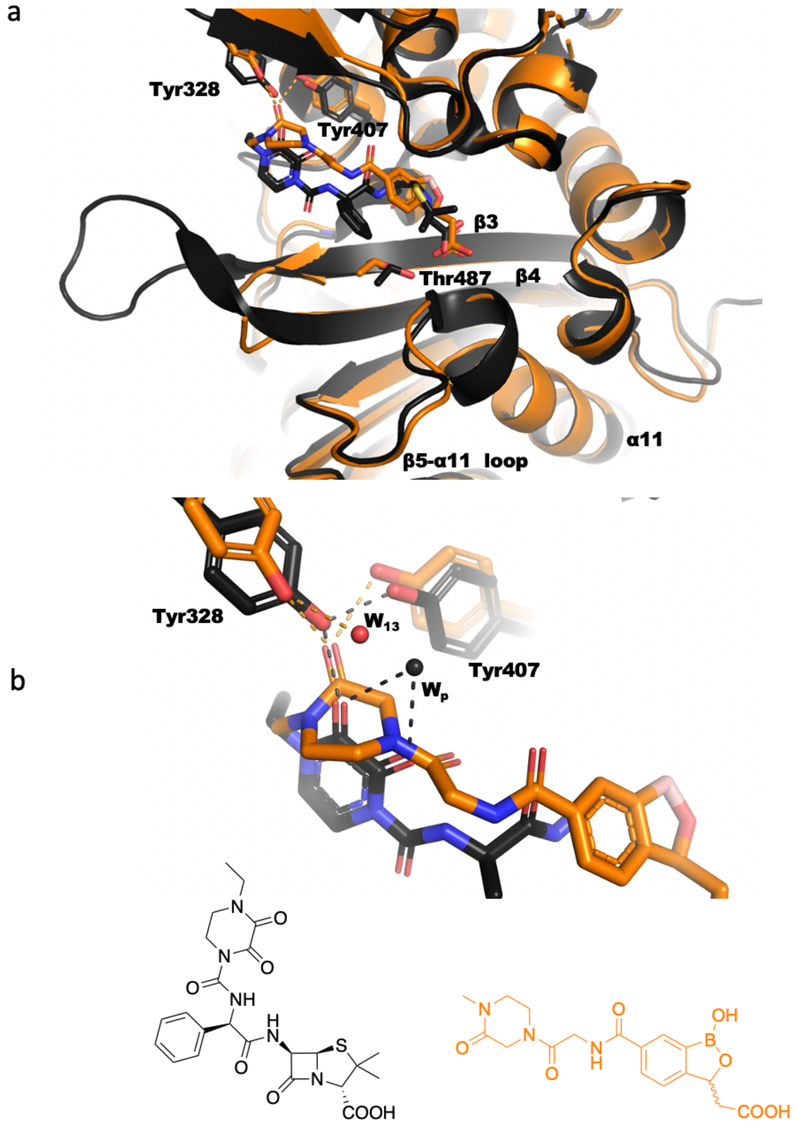

Piperacillin **13**

Figure S13. Comparisons between PaPBP3:13 (orange, PDB: 7AU8) and piperacillin inhibited PaPBP3 (black, PDB: 6R3X)[9] structures. (a) The overall backbone conformation is similar, including for β3 and the β5-α11 loop. Note that the β3-β4 loop is incomplete in the PaPBP3:**13** structure, likely due to conformational flexibility. (b) Interactions of the ketopiperazine group of **13** and the diketopiperazine of reacted piperacillin with Tyr328 and Tyr407. Hydrogen bonds in the PaPBP3**:13** structure are orange dashed lines and are black dashed lines in the piperacillin-reacted PaPBP3 structure. Two waters involved in the hydrogen bonding network of the PaPBP3**:13** structure (**W**$_{13}$) and piperacillin-reacted structure (**W**$_{p}$) are shown.

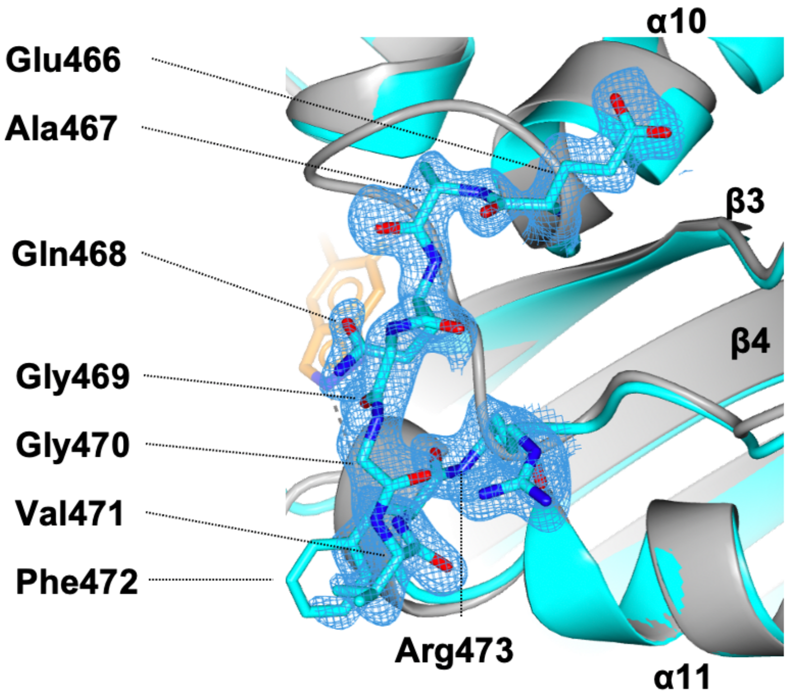

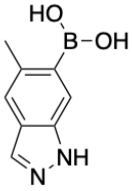

2

Figure S14. Electron density maps for residues 466-473 in the α10-β3 loop which adopt a novel conformation when 2 reacts with PaPBP3 (Figure 7 in the main text). A view from an overlay of the PaPBP3:**2** complex (cyan, PDB: 7ATO) and the piperacillin-reacted structure (gray, PDB: 6R3X).[9] For residues 466-473 of the PaPBP3:**2** complex, each residue and its unbiased omit Fo-Fc map is shown (blue, contoured at 1 σ), as calculated by comit in the ccp4 suite.[21] For the piperacillin-reacted structure, residues 466-473 are in cartoon form. Relevant secondary structure elements are labeled.

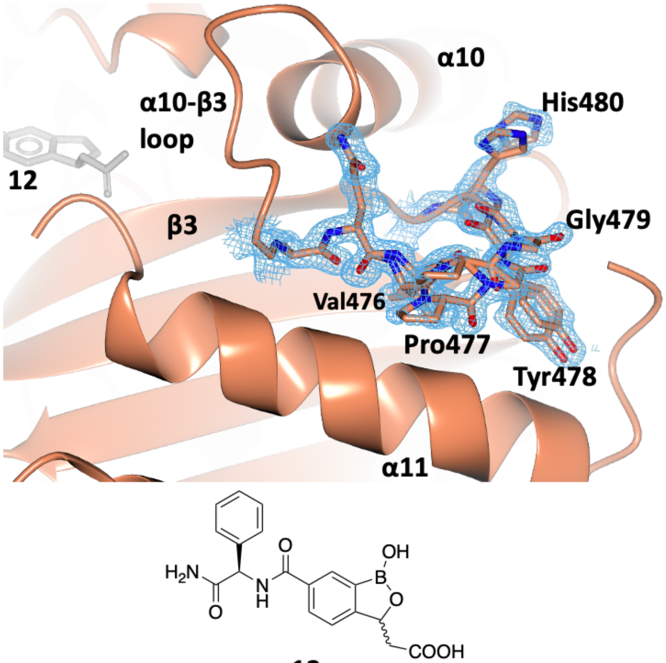

12

Figure S15. Alternate conformations for residues Val476-His480 of the α10-β3 helix in the PaPBP3:12 complex (PDB: 7AU1). Residues with alternate conformations are labeled. All residues were modeled with the same occupancy: 51% for the conformation with residues furthest from α10 and 49% for conformation closest to α10. The unbiased omit Fo-Fc map is shown (contoured at 1 σ), as calculated by comit in the ccp4 suite is shown as blue mesh.[21] Occupancies were determined by refinement using Phenix.[23]

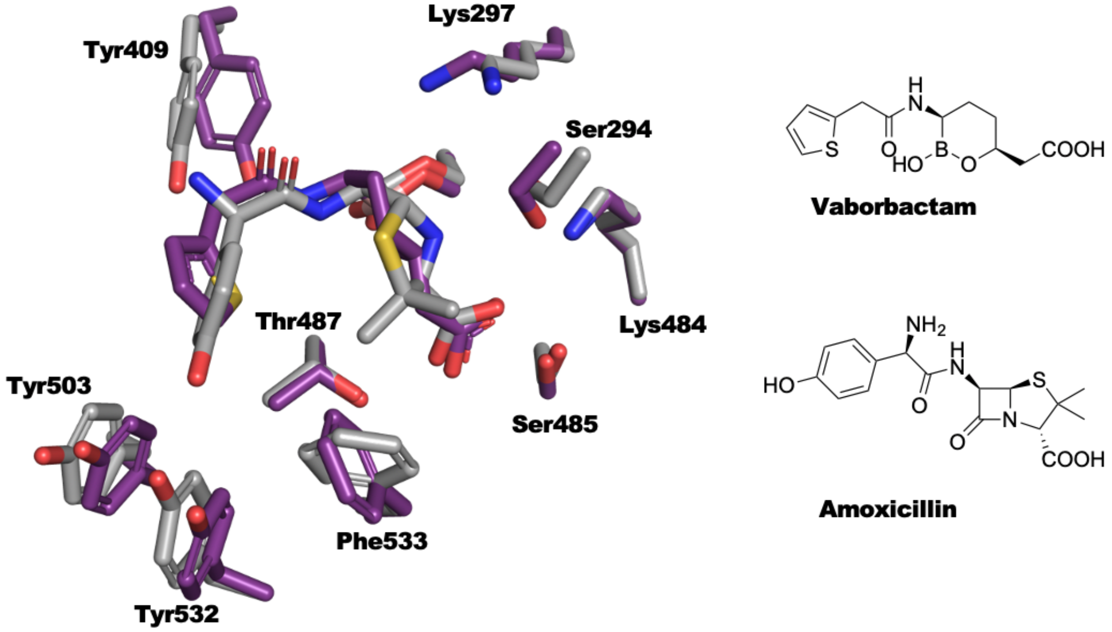

Figure S16. Comparison of the binding modes for Vaborbactam (purple, PDB: 7AUH) and amoxicillin (gray, PDB: 6I1E)[9] with PaPBP3. Neighboring residues are in the same color as the corresponding ligand. The overlay shows considerable alignment of both residue side chain and reacted ligand conformations. Density for Vaborbactam in the PaPBP3:Vaborbactam structure is shown in Figure 8.

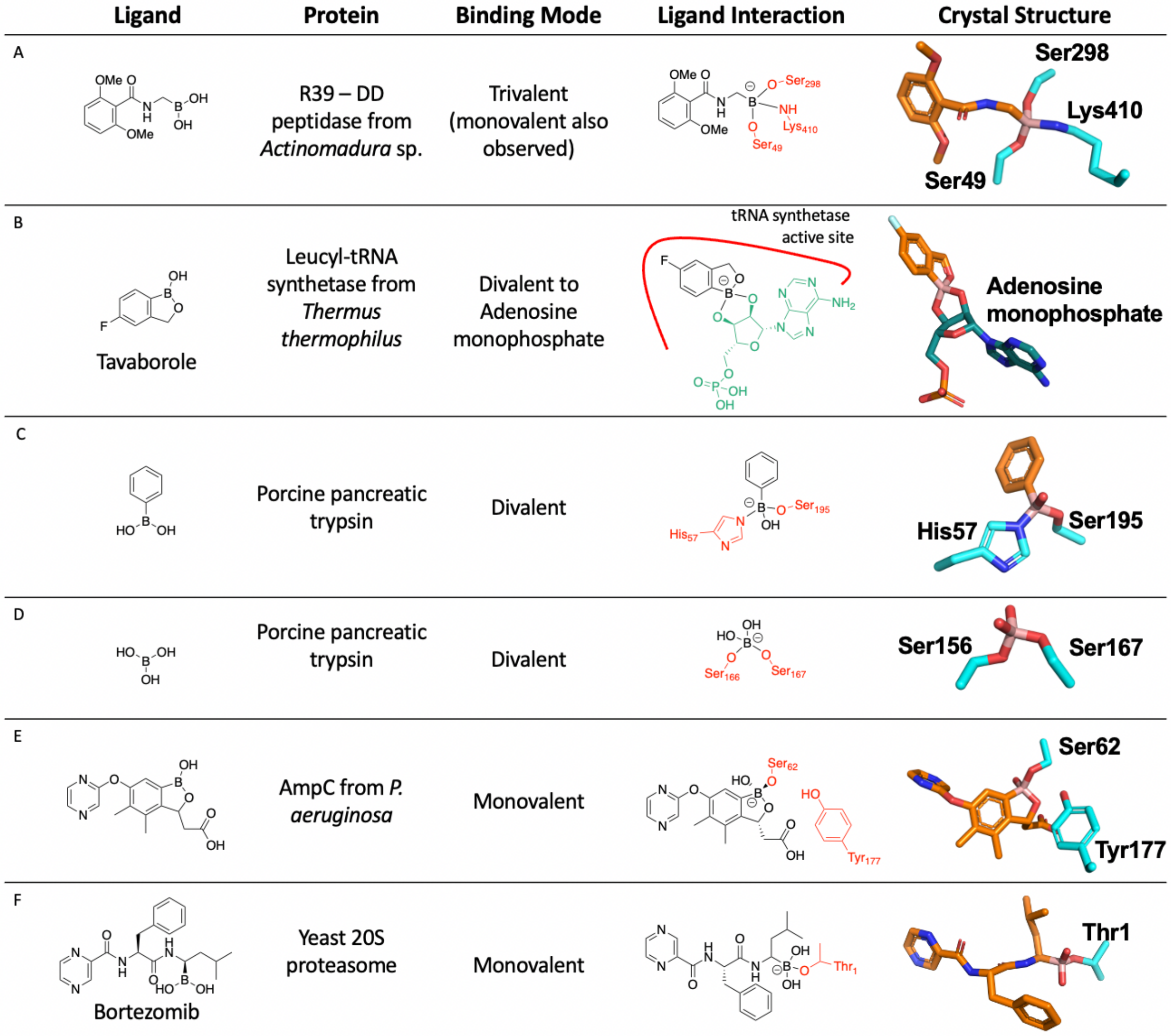

Figure S17. Examples of boron-based inhibition via reaction with nucleophilic residues. (A) An alkyl boronate binding to R39 of *Actinomadura sp*. Aside from our work, this is the only reported example of a tri-covalently-bonded BCI, with analogous nucleophilic residues being involved (PDB: 3ZVT);[20] (B) Di-covalent reaction of a benzoxaborole (the antifungal agent Tavaborole, PDB: 2VOG)[24,25] with the diol ribose moiety of adenosine monophosphate (AMP), in the active site of a leucyl-tRNA synthetase (note the boron does not react with the protein);[24] (C) and (D) example investigations on how simple boronates react with trypsin.[26] Crystal structures imply multiple binding sites for these fragments, with some at lower occupancy (PDB: 2A32 and 2A31, respectively).[26] Only one of the two refined alternate conformations of benzene boronic acid is shown. In the other conformation, the positions of the phenyl and hydroxyl groups are 'switched'; (E) The benzoxaborole scaffold has been employed for inhibition of β-lactamases including AmpC (a class C β-lactamase), which has a related fold to PBP3; however, in AmpC Ser349 of PaPBP3 is replaced by a tyrosine (Tyr177). Di-covalent binding is not observed in this case and the catalytic serine (Ser62) reacts with the opposite face of the benzoxaborole in AmpC compared to direction of attack in PaPBP3 (Figure S18); (F) Bortezomib reacts with a nucleophilic threonine at the proteasome active site (PDB: 2F16).[27]

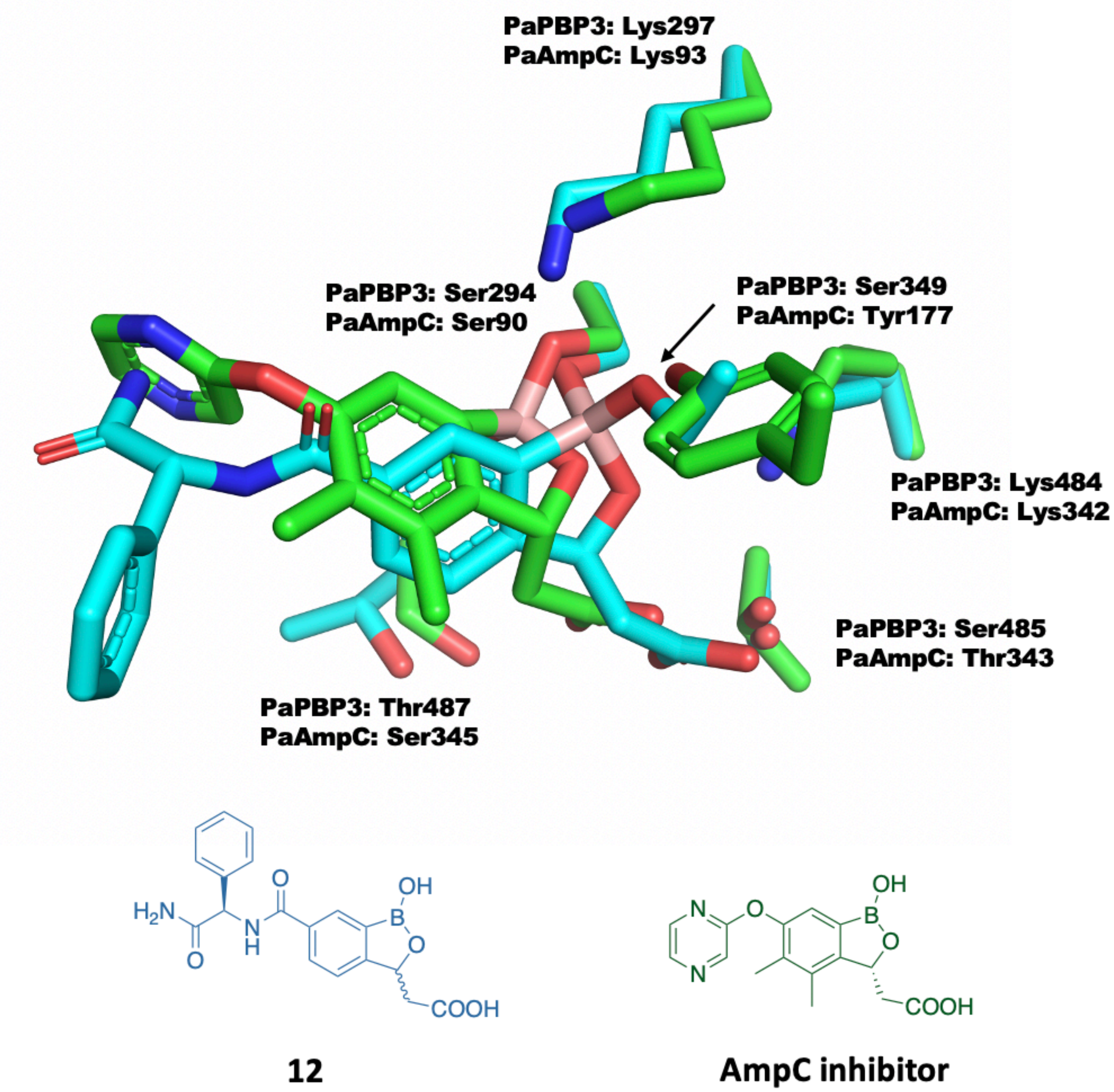

Figure S18. Comparison of the binding mode of benzoxaborole 12 and of a benzoxaborole bound to AmpC.[28] The Cα carbons of active site residues Ser294, Lys297, Ser349 and Lys484 of the PaPBP3:**12** complex (cyan, PDB: 7AU1) were superimposed with equivalent residues in the *P. aeruginosa* AmpC:**benzoxaborole** complex (PaAmpC: Ser90, Lys93, Tyr177 and Lys343, green, PDB: 4WYY).[28] The benzoxaborole is mono-covalently reacted in the PaAmpC complex. The alignment shows that the benzoxaborole core is rotated by ~50° relative to its position in the PaPBP3 structure and that the PaAmpC catalytic serine (Ser90) attacks the other face of the benzoxaborole. The C-3 acid group is positioned similarly in both complexes. Only one of the two alternate conformations of the side chain of PaPBP3 Lys297 is shown for clarity.

HPLC Traces

Compound 5

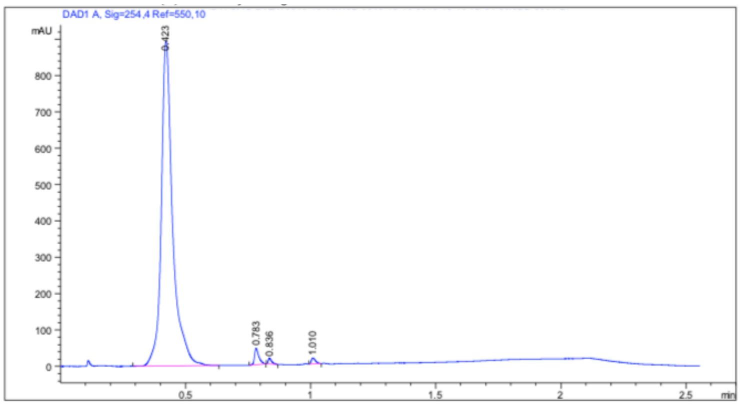

==

Area Percent Report

==

Sorted By : Signal
Multiplier : 1.0000
Dilution : 1.0000
Do not use Multiplier & Dilution Factor with ISTDs

Signal 1: DAD1 A, Sig=254,4 Ref=550,10

Peak #	RetTime [min]	Type	Width [min]	Area [mAU*s]	Height [mAU]	Area %
1	0.423	BB	0.0462	2809.03638	893.39703	96.9137
2	0.783	BB	0.0176	55.13561	45.46098	1.9022
3	0.836	BB	0.0150	15.39992	15.64933	0.5313
4	1.010	BB	0.0174	18.92163	16.47529	0.6528

Totals : 2898.49354 970.98263

6

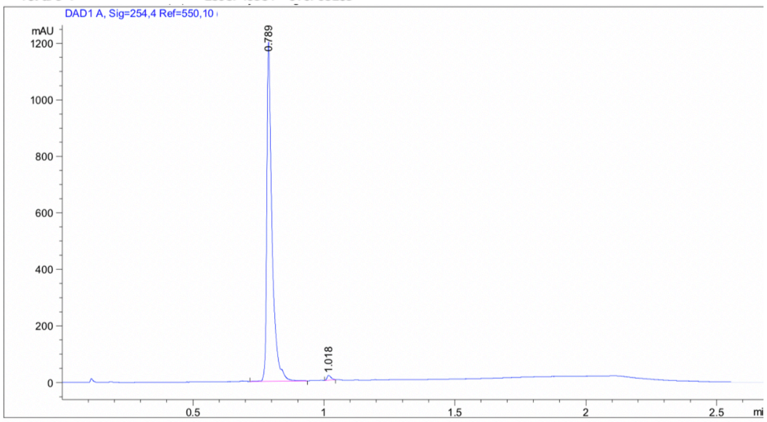

==

Area Percent Report

==

Sorted By : Signal
Multiplier : 1.0000
Dilution : 1.0000
Do not use Multiplier & Dilution Factor with ISTDs

Signal 1: DAD1 A, Sig=254,4 Ref=550,10

Peak #	RetTime [min]	Type	Width [min]	Area [mAU*s]	Height [mAU]	Area %
1	0.789	BB	0.0201	1652.28418	1195.96570	98.9314
2	1.018	BB	0.0162	17.84760	17.04588	1.0686

Totals : 1670.13178 1213.01158

7

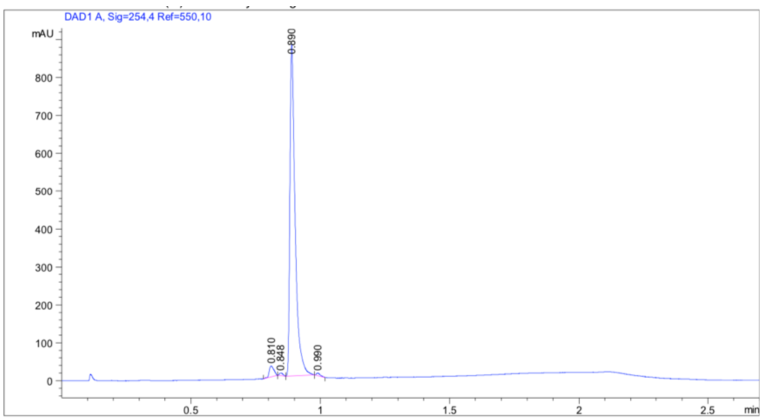


```
=====================================================================
                         Area Percent Report
=====================================================================

Sorted By             :      Signal
Multiplier            :      1.0000
Dilution              :      1.0000
Do not use Multiplier & Dilution Factor with ISTDs


Signal 1: DAD1 A, Sig=254,4 Ref=550,10

Peak RetTime Type  Width     Area      Height     Area
  #   [min]        [min]   [mAU*s]     [mAU]        %
----|-------|----|-------|----------|----------|--------|
   1   0.810 BB    0.0215   37.06830   28.86986   2.8830
   2   0.848 BB    0.0148    6.98682    7.51944   0.5434
   3   0.890 BB    0.0210 1234.62146  874.03674  96.0226
   4   0.990 BB    0.0153    7.08433    7.01685   0.5510

Totals :                  1285.76092  917.44289
```

8

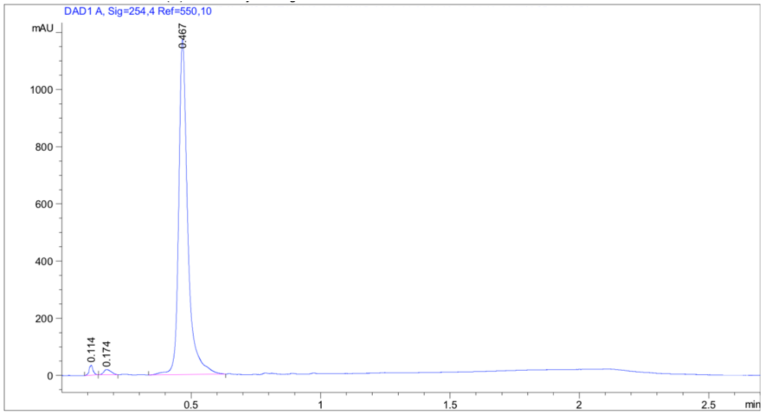


```
=====================================================================
                         Area Percent Report
=====================================================================

Sorted By             :      Signal
Multiplier            :      1.0000
Dilution              :      1.0000
Do not use Multiplier & Dilution Factor with ISTDs


Signal 1: DAD1 A, Sig=254,4 Ref=550,10

Peak RetTime Type  Width     Area      Height     Area
  #   [min]        [min]   [mAU*s]     [mAU]        %
----|-------|----|-------|----------|----------|--------|
   1   0.114 BB    0.0156   35.53299   34.37537   1.2191
   2   0.174 BB    0.0291   35.18584   18.12884   1.2072
   3   0.467 BB    0.0363 2843.91919 1172.33545  97.5737

Totals :                  2914.63802 1224.83966
```

9

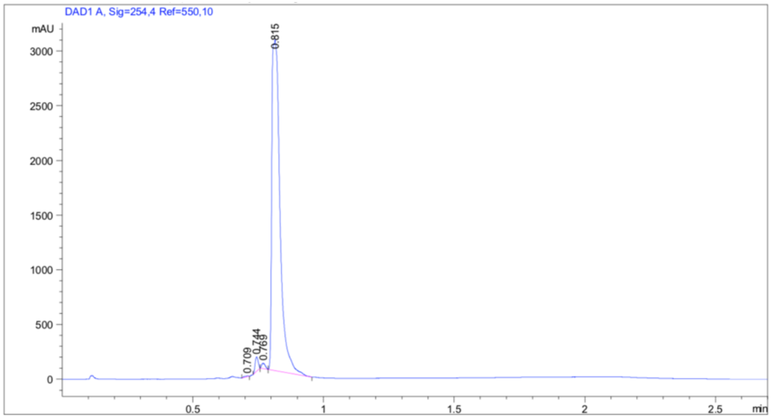

Area Percent Report

Sorted By : Signal
Multiplier : 1.0000
Dilution : 1.0000
Do not use Multiplier & Dilution Factor with ISTDs

Signal 1: DAD1 A, Sig=254,4 Ref=550,10

Peak #	RetTime [min]	Type	Width [min]	Area [mAU*s]	Height [mAU]	Area %
1	0.709	BB	0.0167	9.87181	8.08875	0.1470
2	0.744	BB	0.0127	104.87284	132.30966	1.5612
3	0.769	BB	0.0164	47.70490	48.76843	0.7101
4	0.815	BB	0.0303	6555.15283	3020.34473	97.5817

Totals : 6717.60238 3209.51157

10

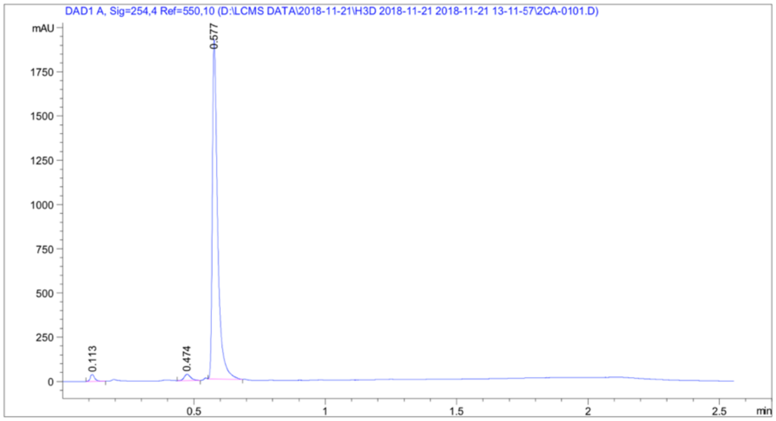

Area Percent Report

Sorted By : Signal
Multiplier : 1.0000
Dilution : 1.0000
Do not use Multiplier & Dilution Factor with ISTDs

Signal 1: DAD1 A, Sig=254,4 Ref=550,10

Peak #	RetTime [min]	Type	Width [min]	Area [mAU*s]	Height [mAU]	Area %
1	0.113	BB	0.0174	43.70873	38.08951	1.5636
2	0.474	BB	0.0254	61.48521	35.98430	2.1995
3	0.577	BB	0.0209	2690.25928	1912.42139	96.2370

Totals : 2795.45322 1986.49519

11

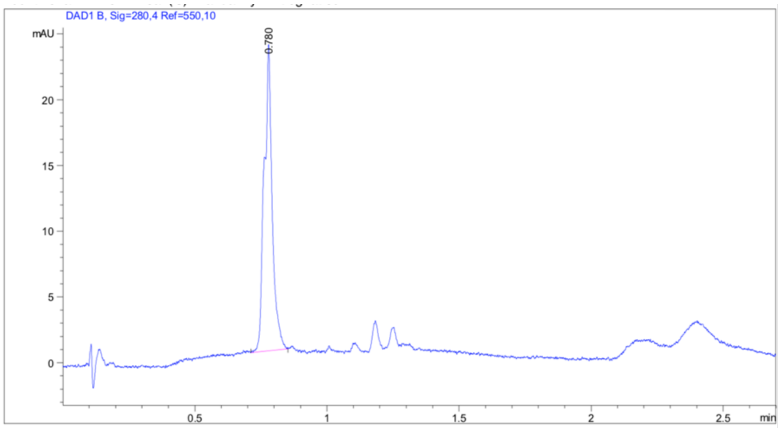

Area Percent Report

Sorted By	:	Signal
Multiplier	:	1.0000
Dilution	:	1.0000

Do not use Multiplier & Dilution Factor with ISTDs

Signal 1: DAD1 B, Sig=280,4 Ref=550,10

Peak #	RetTime [min]	Type	Width [min]	Area [mAU*s]	Height [mAU]	Area %
1	0.780	BB	0.0283	49.37632	23.25029	100.0000

Totals : 49.37632 23.25029

14

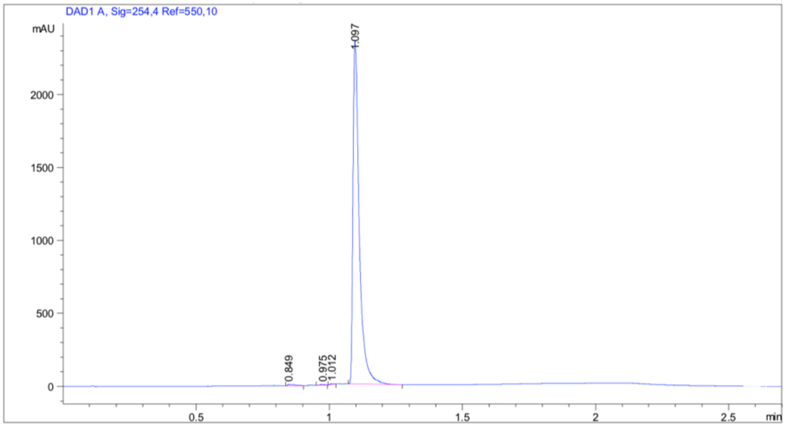

Area Percent Report

Sorted By	:	Signal
Multiplier	:	1.0000
Dilution	:	1.0000

Do not use Multiplier & Dilution Factor with ISTDs

Signal 1: DAD1 A, Sig=254,4 Ref=550,10

Peak #	RetTime [min]	Type	Width [min]	Area [mAU*s]	Height [mAU]	Area %
1	0.849	BB	0.0232	16.37372	9.21772	0.4202
2	0.975	BB	0.0166	5.93035	5.48081	0.1522
3	1.012	BB	0.0165	5.44125	5.51068	0.1396
4	1.097	BB	0.0247	3868.62329	2355.69897	99.2879

Totals : 3896.36861 2375.90818

15

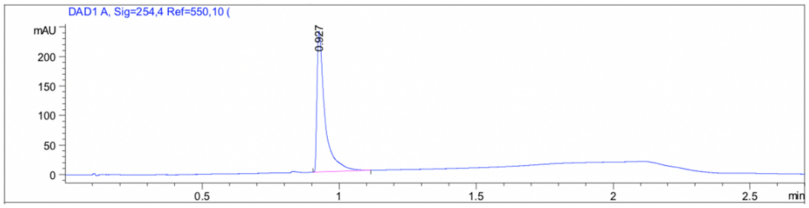


```
=====================================================================
                         Area Percent Report
=====================================================================

Sorted By             :      Signal
Multiplier            :      1.0000
Dilution              :      1.0000
Do not use Multiplier & Dilution Factor with ISTDs


Signal 1: DAD1 A, Sig=254,4 Ref=550,10

Peak RetTime Type  Width     Area      Height     Area
  #   [min]        [min]   [mAU*s]     [mAU]        %
----|-------|----|-------|----------|----------|--------|
   1   0.927 BB    0.0290  480.42749  238.69498 100.0000

Totals :                   480.42749  238.69498
```

References

(1) Krishnamoorthy, G.; Wolloscheck, D.; Weeks, J. W.; Croft, C.; Rybenkov, V. V.; Zgurskaya, H. I. Breaking the Permeability Barrier of *Escherichia Coli* by Controlled Hyperporination of the Outer Membrane. *Antimicrob. Agents Chemother.* **2016**.

(2) Dougherty, P. F.; Yotter, D. W.; Matthews, T. R. Microdilution Transfer Plate Technique for Determining In Vitro Synergy of Antimicrobial Agents. *Antimicrob. Agents Chemother.* **1977**, *11* (2), 225–228.

(3) De Meester, F.; Joris, B.; Reckinger, G.; Bellefroid-Bourguignon, C.; Frère, J.-M.; Waley, S. G. Automated Analysis of Enzyme Inactivation Phenomena. *Biochem. Pharmacol.* **1987**, *36* (14), 2393–2403.

(4) Brem, J.; Cain, R.; Cahill, S.; McDonough, M. A.; Clifton, I. J.; Jiménez-Castellanos, J.-C.; Avison, M. B.; Spencer, J.; Fishwick, C. W. G.; Schofield, C. J. Structural Basis of Metallo-β-Lactamase, Serine-β-Lactamase and Penicillin-Binding Protein Inhibition by Cyclic Boronates. *Nat. Commun.* **2016**, *7* (1), 12406.

(5) Graves-Woodward, K.; Pratt, R. F. Reaction of Soluble Penicillin-Binding Protein 2a of Methicillin-Resistant Staphylococcus Aureus with β-Lactams and Acyclic Substrates: Kinetics in Homogeneous Solution. *Biochem. J.* **1998**, *332* (3), 755–761.

(6) Berthold, M. R.; Cebron, N.; Dill, F.; Gabriel, T. R.; Kötter, T.; Meinl, T.; Ohl, P.; Thiel, K.; Wiswedel, B. KNIME - the Konstanz Information Miner: Version 2.0 and Beyond. *ACM SIGKDD Explor. Newsl.* **2009**, *11* (1), 26–31.

(7) Ashton, M.; Barnard, J.; Casset, F.; Charlton, M.; Downs, G.; Gorse, D.; Holliday, J.; Lahana, R.; Willett, P. Identification of Diverse Database Subsets Using Property-Based and Fragment-Based Molecular Descriptions. *Quant. Struct.-Act. Relatsh.* **2002**, *21* (6), 598–604.

(8) *RDKit: Open-Source Cheminformatics;*

(9) Bellini, D.; Koekemoer, L.; Newman, H.; Dowson, C. G. Novel and Improved Crystal Structures of H. Influenzae, E. Coli and P. Aeruginosa Penicillin-Binding Protein 3 (PBP3) and N. Gonorrhoeae PBP2: Toward a Better Understanding of β-Lactam Target-Mediated Resistance. *J. Mol. Biol.* **2019**, *431* (18), 3501–3519.

(10) Johnson, K. A. New Standards for Collecting and Fitting Steady State Kinetic Data. *Beilstein J. Org. Chem.* **2019**, *15*, 16–29.

(11) Shapiro, A. B.; Gu, R.-F.; Gao, N.; Livchak, S.; Thresher, J. Continuous Fluorescence Anisotropy-Based Assay of BOCILLIN FL Penicillin Reaction with Penicillin Binding Protein 3. *Anal. Biochem.* **2013**, *439* (1), 37–43.

(12) Tickle, I. J.; Flensburg, C.; Keller, P.; Paciorek, W.; Sharff, A.; Vonrhein, C.; Bricogne, G. *STARANISO*; Global Phasing Ltd.: Cambridge, United Kingdom.

(13) Dong, H.; Zhang, Z.; Tang, X.; Paterson, N. G.; Dong, C. Structural and Functional Insights into the Lipopolysaccharide ABC Transporter LptB2FG. *Nat. Commun.* **2017**, *8* (1), 222.

(14) Vonrhein, C.; Flensburg, C.; Keller, P.; Sharff, A.; Smart, O.; Paciorek, W.; Womack, T.; Bricogne, G. Data Processing and Analysis with the *AutoPROC* Toolbox. *Acta Crystallogr. D Biol. Crystallogr.* **2011**, *67* (4), 293–302.

(15) Sainsbury, S.; Bird, L.; Rao, V.; Shepherd, S. M.; Stuart, D. I.; Hunter, W. N.; Owens, R. J.; Ren, J. Crystal Structures of Penicillin-Binding Protein 3 from Pseudomonas Aeruginosa: Comparison of Native and Antibiotic-Bound Forms. *J. Mol. Biol.* **2011**, *405* (1), 173–184.

(16) Bowler, L. D.; Spratt, B. G. Membrane Topology of Penicillin-Binding Protein 3 of Escherichia Coli. *Mol. Microbiol.* **1989**, *3* (9), 1277–1286.

(17) Pares, S.; Mouz, N.; Pétillot, Y.; Hakenbeck, R.; Dideberg, O. X-Ray Structure of Streptococcus Pneumoniae PBP2x, a Primary Penicillin Target Enzyme. *Nat. Struct. Biol.* **1996**, *3* (3), 284–289.

(18) Madeira, F.; Park, Y. mi; Lee, J.; Buso, N.; Gur, T.; Madhusoodanan, N.; Basutkar, P.; Tivey, A. R. N.; Potter, S. C.; Finn, R. D.; Lopez, R. The EMBL-EBI Search and Sequence Analysis Tools APIs in 2019. *Nucleic Acids Res.* **2019**, *47* (W1), W636–W641.
(19) Robert, X.; Gouet, P. Deciphering Key Features in Protein Structures with the New ENDscript Server. *Nucleic Acids Res.* **2014**, *42* (W1), W320–W324.
(20) Zervosen, A.; Herman, R.; Kerff, F.; Herman, A.; Bouillez, A.; Prati, F.; Pratt, R. F.; Frère, J.-M.; Joris, B.; Luxen, A.; Charlier, P.; Sauvage, E. Unexpected Tricovalent Binding Mode of Boronic Acids within the Active Site of a Penicillin-Binding Protein. *J. Am. Chem. Soc.* **2011**, *133* (28), 10839–10848.
(21) Winn, M. D.; Ballard, C. C.; Cowtan, K. D.; Dodson, E. J.; Emsley, P.; Evans, P. R.; Keegan, R. M.; Krissinel, E. B.; Leslie, A. G. W.; McCoy, A.; McNicholas, S. J.; Murshudov, G. N.; Pannu, N. S.; Potterton, E. A.; Powell, H. R.; Read, R. J.; Vagin, A.; Wilson, K. S. Overview of the *CCP* 4 Suite and Current Developments. *Acta Crystallogr. D Biol. Crystallogr.* **2011**, *67* (4), 235–242.
(22) Han, S.; Zaniewski, R. P.; Marr, E. S.; Lacey, B. M.; Tomaras, A. P.; Evdokimov, A.; Miller, J. R.; Shanmugasundaram, V. Structural Basis for Effectiveness of Siderophore-Conjugated Monocarbams against Clinically Relevant Strains of Pseudomonas Aeruginosa. *Proc. Natl. Acad. Sci.* **2010**, *107* (51), 22002–22007.
(23) Adams, P. D.; Afonine, P. V.; Bunkóczi, G.; Chen, V. B.; Davis, I. W.; Echols, N.; Headd, J. J.; Hung, L.-W.; Kapral, G. J.; Grosse-Kunstleve, R. W.; McCoy, A. J.; Moriarty, N. W.; Oeffner, R.; Read, R. J.; Richardson, D. C.; Richardson, J. S.; Terwilliger, T. C.; Zwart, P. H. *PHENIX* : A Comprehensive Python-Based System for Macromolecular Structure Solution. *Acta Crystallogr. D Biol. Crystallogr.* **2010**, *66* (2), 213–221.
(24) Rock, F. L.; Mao, W.; Yaremchuk, A.; Tukalo, M.; Crepin, T.; Zhou, H.; Zhang, Y.-K.; Hernandez, V.; Akama, T.; Baker, S. J.; Plattner, J. J.; Shapiro, L.; Martinis, S. A.; Benkovic, S. J.; Cusack, S.; Alley, M. R. K. An Antifungal Agent Inhibits an Aminoacyl-TRNA Synthetase by Trapping TRNA in the Editing Site. *Science* **2007**, *316* (5832), 1759–1761.
(25) Sharma, N.; Sharma, D. An Upcoming Drug for Onychomycosis: Tavaborole. *J. Pharmacol. Pharmacother.* **2015**, *6* (4), 236.
(26) Transue, T. R.; Gabel, S. A.; London, R. E. NMR and Crystallographic Characterization of Adventitious Borate Binding by Trypsin. *Bioconjug. Chem.* **2006**, *17* (2), 300–308.
(27) Groll, M.; Berkers, C. R.; Ploegh, H. L.; Ovaa, H. Crystal Structure of the Boronic Acid-Based Proteasome Inhibitor Bortezomib in Complex with the Yeast 20S Proteasome. *Structure* **2006**, *14* (3), 451–456.
(28) McKinney, D. C.; Zhou, F.; Eyermann, C. J.; Ferguson, A. D.; Prince, D. B.; Breen, J.; Giacobbe, R. A.; Lahiri, S.; Verheijen, J. C. 4,5-Disubstituted 6-Aryloxy-1,3-Dihydrobenzo[*c*][1,2]Oxaboroles Are Broad-Spectrum Serine β-Lactamase Inhibitors. *ACS Infect. Dis.* **2015**, *1* (7), 310–316.
